# Topological Vulcanization Strategy for Elastomeric Electrolytes with Enhanced Mechanical and Electrochemical Properties for Advanced Lithium Metal Batteries

**DOI:** 10.1002/advs.202506640

**Published:** 2025-07-14

**Authors:** Na Yang, Haotian Meng, DeCai Guo, Yongyi Song, Yongzheng Shi, Jin Niu, Feng Wang

**Affiliations:** ^1^ State Key Laboratory of Chemical Resource Engineering Beijing Key Laboratory of Electrochemical Process and Technology for Materials Beijing University of Chemical Technology Beijing 100029 P. R. China; ^2^ National Engineering Research Center for Fuel Cell and Hydrogen Source Technology Beijing University of Chemical Technology Beijing 100029 P. R. China; ^3^ Section 14, Dalian Research Institute of Petroleum and Petrochemicals SINOPEC, No.96 Nankai Road, Tieshan Street, Lvshunkou District, Dalian, Liaoning Province P. R. China; ^4^ College of Environmental Science and Engineering North China Electric Power University Beijing 102206 P. R. China

**Keywords:** elastomeric electrolytesï, solid‐state lithium metal battery, topological vulcanization

## Abstract

Elastomeric electrolytes (EEs) have garnered significant attention in the realm of next‐generation energy storage systems, attributed to their distinctive mechanical properties. Nonetheless, achieving precise modulation of mechanical robustness while ensuring efficient lithium‐ion transport continues to present a significant challenge. In this study, an innovative topological vulcanization strategy is proposed to fabricate uniformly crosslinked EEs. The resultant EEs demonstrate exceptional mechanical properties, characterized by a tensile strength of 3.51 MPa and an elongation at break of 832%, alongside a room‐temperature ionic conductivity of 4 × 10^−4^ S cm^−1^. Such robust and flexible electrolytes maintain superior structural integrity throughout battery operation. This approach enables stable cycling for more than 3000 h in lithium symmetric cells and achieves 95.7% capacity retention after 500 cycles at a rate of 0.5C in full cells. They also exhibit consistent and dependable performance in stretchable and pouch cells even under significant deformations, thereby confirming their suitability for advanced flexible energy storage applications.

## Graphical abstract

Elastomeric electrolytes (EEs) have garnered significant attention in the realm of energy storage system, attributed to their distinctive mechanical properties. In this study, we propose an innovative topological vulcanization stategy to fabricate uniformly crosslinked EEs. The resultant EEs demonstate exceptional mechanical and electrochemical properties. The EEs have been successfully applied in stretchable batteries, provideing a solution for the energy supply of flexible devices.

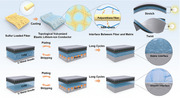

## 1 Introduction

The increasing demand for efficient energy conversion and storage systems has substantially accelerated the development of rechargeable batteries with superior performance and enhanced safety.^[^ ^1^, ^2^, ^3^ ^]^ Among these, lithium‐based batteries are widely recognized as being crucial for the advancement of clean energy technologies. Nevertheless, commercial lithium‐ion batteries are nearing their theoretical energy density ceiling as a result of the limited capacity of graphite anodes.^[^ ^4^ ^]^ Moreover, safety concerns have been significantly amplified by the potential hazards associated with the leakage and flammability of organic liquid electrolytes.^[^ ^2^, ^5^ ^]^ Solid‐state lithium metal batteries (SSLMBs) have emerged as promising candidates due to their higher energy density, enhanced safety, and superior adaptability for specialized applications such as wearable, stretchable, and implantable devices.^[^ ^6^, ^7^ ^]^ By replacing flammable liquid electrolytes with their solid‐state counterparts and utilizing metallic lithium as an anode, SSLMBs overcome key limitations of commercial lithium‐ion batteries, enabling next‐generation energy storage solutions.^[^ ^8^ ^]^

Solid‐state electrolytes, as a crucial component of SSLMBs, can be classified into three main categories: solid polymer electrolytes,^[^ ^9^ ^]^ inorganic solid electrolytes,^[^ ^10^ ^]^ and their composites.^[^ ^11^, ^12^ ^]^ Each type possesses unique advantages and limitations. Achieving sufficient ionic conductivities (typically exceeding 10^−4^ S cm^−1^ at room temperature) and wide electrochemical stability windows (for instance, within the range of 4–5 V) is a fundamental requirement.^[^ ^6^ ^]^ Furthermore, the mechanical properties of solid‐state electrolytes, including compressive strength, fracture toughness, and resilience, are of equal importance.^[^ ^13^ ^]^ These properties are intrinsically linked to the operational safety and cycle life of SSLMBs, especially under conditions involving repeated deformation or mechanical stress.^[^ ^14^, ^15^ ^]^ Among the available options, elastomeric electrolytes with superior resilience have been identified as a particularly promising solution compared to brittle inorganic solid electrolytes and conventional polymer‐based electrolytes, such as polyethylene oxide and polyvinylidene fluoride. The intrinsic elastic properties of elastomers not only enhance their capability to accommodate volume changes during lithium plating and stripping but also offer superior adaptability for advanced applications.^[^ ^16^ ^]^

To integrate mechanical resilience with electrochemical functionality, numerous methodologies have been devised to optimize elastomeric electrolytes for SSLMBs. For instance, Cui et al.^[^ ^17^ ^]^ introduced a dual‐crosslinking strategy to develop highly resilient lithium‐ion conductors. By incorporating dynamic hydrogen bonds, the fabricated electrolytes demonstrated excellent resilience of 0.32 MJ m^−3^ while maintaining a moderate ionic conductivity of 2.5 × 10^−4^ S cm^−1^. Subsequently, Kim et al.^[^ ^18^, ^19^, ^20^ ^]^ successfully developed a series of elastomeric electrolytes through an innovative polymerization‐induced phase separation strategy. By optimizing the ratio of elastomer to plastic crystal phases, the resultant plastic crystal‐embedded elastomeric electrolytes achieved an elongation at break exceeding 300% along with specialized features, such as high‐voltage resistance and low‐temperature performance. In our previous studies,^[^ ^21^, ^22^ ^]^ we developed a series of elastomeric electrolytes based on nitrile butadiene rubber (NBR) by modulating the extent of vulcanization. This approach yielded electrolytes exhibiting superior electrochemical performance, alongside an enhanced resilience of 0.92 MJ m^−3^. Regardless of the synthesis strategy utilized, the elastic properties of elastomeric electrolytes are primarily determined by the crosslink density within their polymer matrices, which also significantly influences ionic conductivity.^[^ ^23^ ^]^ Generally, an increased crosslink density enhances mechanical strength while impeding ion transport. Therefore, achieving an optimal balance between these competing requirements via precise and controllable regulation remains a significant challenge in the development of high‐performance elastomeric electrolytes.

In this study, we introduce an innovative topological vulcanization strategy aimed at developing elastomeric electrolytes that concurrently exhibit enhanced mechanical strength and improved lithium‐ion transport properties. By integrating sulfur‐containing polyurethane fibers into the vulcanization process of a chemically modified NBR matrix, the resultant topological vulcanized elastomeric electrolytes (TVEEs) can be precisely engineered to achieve adjustable levels of elasticity and Young's modulus. The as‐prepared TVEEs demonstrate uniform crosslinking, resulting in a distinctive combination of superior elasticity (with elongation at break reaching up to 832%), remarkable mechanical strength (with stress up to 3.51 MPa), and high ionic conductivity (4 × 10^−4^ S cm^−1^) at room temperature. Consequently, the TVEEs exhibit superior interfacial compatibility with metallic lithium anodes, thereby enabling a significantly extended cycle life of 3000 h in lithium symmetric cells and maintaining 95.7% capacity retention after 500 cycles at 0.5C in full cells. Furthermore, the TVEEs were effectively utilized in stretchable batteries and pouch cells, showcasing their dependable performance under significant deformations and exceptional safety characteristics.

## 2 Results and Discussion

**Scheme 1:**
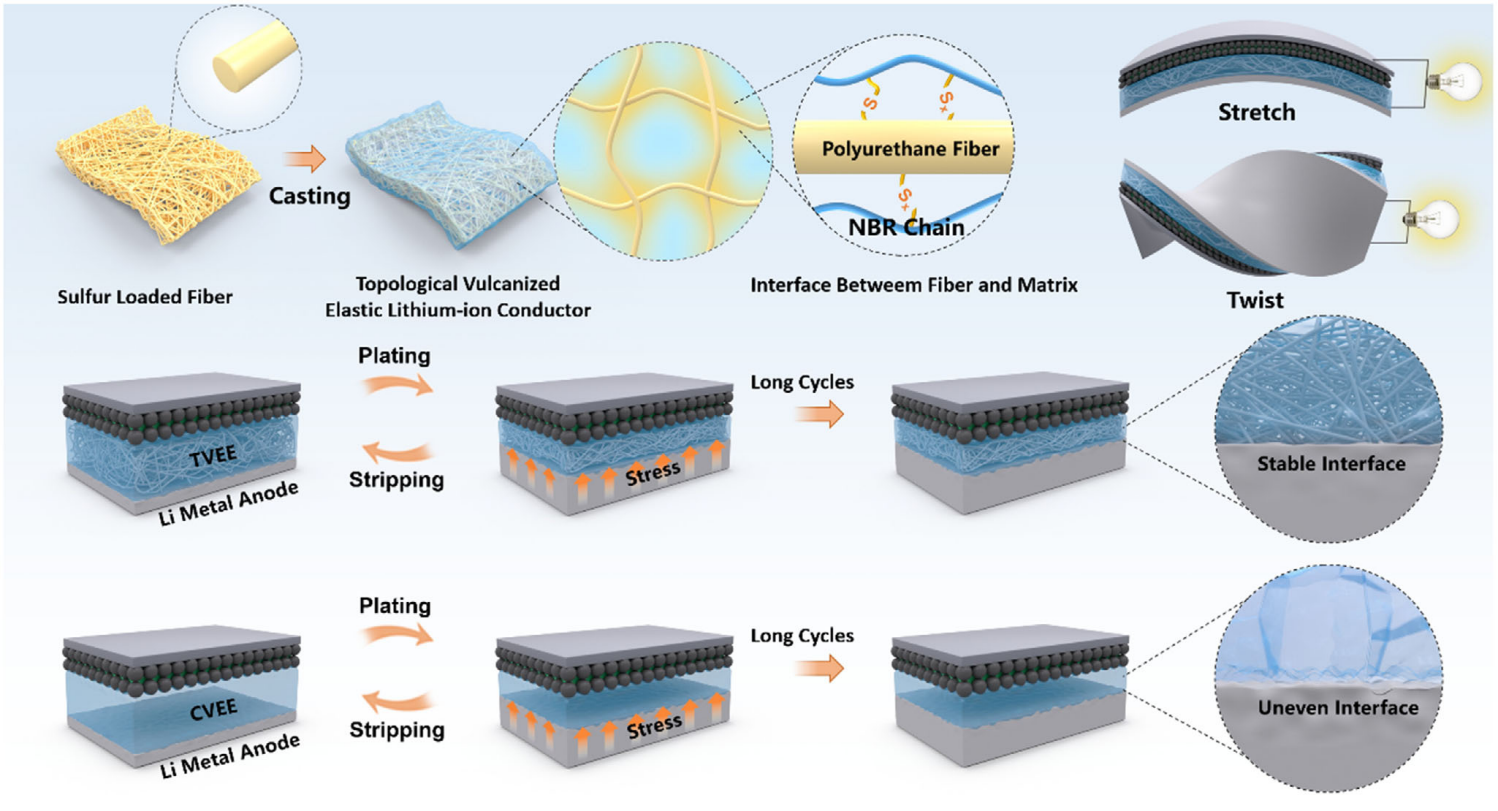
Schematic illustration of TVEE fabrication process and electrochemical‐mechanical performance assessment.

The topological vulcanization strategy is demonstrated by utilizing sulfur‐containing polyurethane fibers as vulcanizing agent carriers for the in situ vulcanization of a chemically modified nitrile butadiene rubber (NBR) matrix at 180 °C (Scheme 1). The fibers were fabricated via the electrospinning of a homogeneous solution consisting of polyurethane and adjustable concentrations of sulfur dissolved in tetrahydrofuran (as shown in Figure S1, Supporting Information). The topological vulcanized elastomeric electrolyte (TVEE) is synthesized via a three‐step procedure: first, a mixed solution of acetone and xylene containing chemically modified NBR is cast onto a polyurethane fiber membrane. Polyurethane was selected as a mechanically robust and elastic scaffold owing to its excellent electrospinnability, mechanical flexibility, and thermal stability (Figure S2, Supporting Information). Second, the assembly undergoes vacuum drying; and third, in situ vulcanization is performed at an elevated temperature. Prior to casting, the NBR undergoes chemical modification by incorporating carbonyl‐rich 4‐vinyl‐1,3‐dioxolan‐2‐one and fluorine‐rich pentafluorostyrene in a mix solution of acetone and xylene. As shown in Figures S3 and S4 (Supporting Information), new peaks emerge in of both conventional vulcanized elastomeric electrolyte (CVEE) and TVEE samples at 1327 cm^−1^, corresponding to the characteristic C═O stretching vibration of VEC, confirming the successful grafting of VEC. Notably, the enlarged view reveals a noticeable shift in the C≡N characteristic peak in both TVEE and CVEE samples, along with the appearance of a distinct ‐S‐C≡N signal at 2164 cm^−1^ in the TVEE sample, indicating the effective vulcanization process (Figure S4, Supporting Information).

These polar functional groups are anticipated to enhance the dissociation of lithium salts through Lewis acid‐base interactions, thereby facilitating lithium‐ion transport within the TVEE. Scanning electron microscopy (SEM) images and corresponding energy dispersive spectroscopy (EDS) mapping of the TVEE membrane confirm the homogeneous distribution of polyurethane fibers within the NBR matrix (Figures S5 and S6, Supporting Information). For comparison, a CVEE was prepared by incorporating an equivalent amount of sulfur directly into the NBR matrix under identical vulcanization conditions.

**Figure 1:**
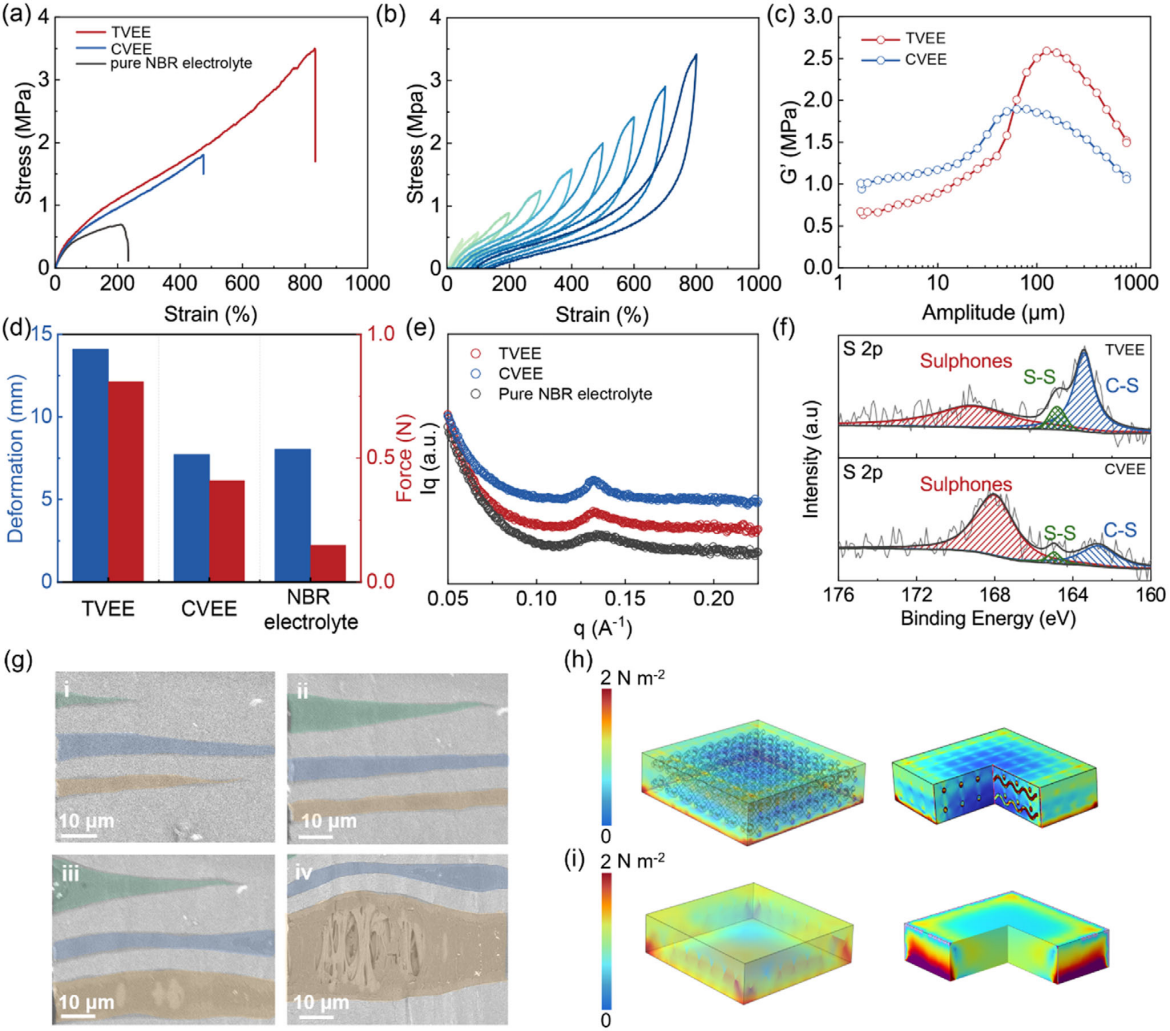
Mechanical and structural analyses of elastomeric electrolytes. a) Stress‐strain curves of the TVEE, CVEE, and pure NBR electrolyte. b) Stress‐strain cycling curves of the TVEE membrane at various strains. c) Dynamic thermo‐mechanical analysis of the TVEE and CVEE membrane. d) Force‐displacement diagrams and e) X‐ray small‐angle scattering patterns of the TVEE, CVEE, and pure NBR electrolyte. f) XPS spectra of S 2p for the TVEE and CVEE membrane. g) In situ SEM images of the TVEE under tensile stress. COMSOL simulations of the h) TVEE and i) NBR electrolyte membranes under applied stress.

Reliable mechanical properties of the as‐prepared NBR‐based electrolytes are crucial for ensuring cycling stability, safety, and adaptability across a wide range of applications. The stress‐strain curve presented in **Figure** **1**a demonstrates the exceptional mechanical properties of the TVEE membrane. Notably, the TVEE membrane demonstrates outstanding mechanical properties, characterized by a tensile stress of 3.51 MPa and an elongation at a break of 832%, which is highly remarkable. These values are considerably higher than those of the CVEE membrane, which exhibits a tensile stress of 1.8 MPa and an elongation at break of 474%. It is important to highlight that the pure NBR electrolyte exhibits extremely low tensile stress and elongation due to the absence of a vulcanization process. Furthermore, the TVEE membrane demonstrates exceptional resilient properties, fully recovering its original state even when subjected to a high strain of 800% (as shown in Figure 1b; Figure S7, Supporting Information). This stands in stark contrast to the CVEE membrane, which exhibits a maximum recoverable strain of 350% with stress of 1.18 MPa, which is significantly lower than that of TVEE. (Figure S8, Supporting Information). This abrupt reduction in stress is likely attributable to the detachment between the polyurethane skeleton and the polymer matrix under high‐strain conditions. What's more, adjusting the sulfur content of polyurethane fibers enables precise control over their mechanical properties (Figures S9 and S10, Supporting Information). To further assess the mechanical properties of the TVEE membranes, the storage modulus was evaluated through dynamic mechanical analysis under controlled strain conditions. As depicted in Figure 1c, the TVEE membrane demonstrates a substantially higher storage modulus at larger strain amplitudes in comparison to the CVEE membrane, indicating an enhanced ability to accommodate the volume changes during cycling and to prevent dendrite penetration. Additionally, the TVEE membrane exhibits superior mechanical robustness and viscoelastic balance compared to the CVEE membrane. The higher storage modulus and loss modulus confirm that the topological vulcanization strategy effectively enhances both the elastic strength and energy dissipation ability of the electrolyte (Figure S11, Supporting Information). The enhanced robustness and resilience of the system can be primarily attributed to the implementation of the topological vulcanization strategy. As shown in Figure 1d, the penetration resistance of the prepared electrolyte was tested. Owing to the highly efficient topological vulcanization, the TVEE demonstrates a significantly enhanced penetration force, nearly double that of the CVEE. To elucidate the molecular structure of the TVEE electrolyte and to reveal the mechanism of topological vulcanization, small‐angle X‐ray scattering (SAXS) and X‐ray photoelectron spectroscopy (XPS) were employed. As depicted in Figure 1e, the TVEE membrane exhibits a broad peak, indicating a uniform distribution of sulfur bonds located at the interface throughout the electrolyte.^[^ ^24^, ^25^ ^]^ In contrast, the CVEE membrane exhibits a more pronounced sharp peak, which is indicative of non‐uniform vulcanization and an increase in local crystallinity. This enhancement in crystallinity not only compromises the mechanical properties but also diminishes the ionic conductivity of NBR‐based electrolytes.

Further chemical analysis, as shown in Figure 1f, presents the high‐resolution S 2p spectrum, which can be deconvoluted into three distinct peaks. These peaks are attributed to C─S bonds, indicating effectively incorporated sulfur within the polymer matrix through covalent interactions^[^ ^26^ ^]^; S─S bonds, representing disulfide linkages and residual sulfur within the matrix;^[^ ^27^ ^]^ and sulfones, corresponding to short‐chain organosulfides.^[^ ^26^, ^28^ ^]^ In the vulcanization process, the sulfur within the polyurethane fibers reacts with the unsaturated double bonds (─C═C─) of the NBR matrix, forming covalent C–S_(‐x‐)_–C crosslinks. The TVEE membrane exhibits a markedly enhanced C‐S peak, with a peak area ratio of 45.5%, significantly higher than that of the CVEE membrane (24.2%). This indicates a higher extent of sulfur incorporation into the polymer matrix. In contrast, the CVEE membrane shows a more pronounced sulfone peak (with a peak area ratio of 71.7%), indicating a higher proportion of sulfur in oxidized states due to direct vulcanization (Figure S12, Supporting Information). The enhanced S–S and C–S signals observed in the TVEE membrane indicate the superior crosslinking efficiency achieved via topological vulcanization. This process facilitates more efficient and controllable sulfur incorporation into the NBR‐based network, in contrast to the lower efficiency and uniformity observed in conventional vulcanization.

The enhanced mechanical properties of the TVEE membrane reveal the critical role of topological vulcanization, which is initiated by sulfur locally delivered via electrospun polyurethane fibers acting as a topological scaffold. These fibers define the spatial distribution of crosslinking sites, promoting the formation of interconnected yet locally confined polymer networks. The controlled release of the vulcanizing agent promotes the formation of a robust crosslinking network and enhances interfacial bonding between polyurethane fibers and the NBR matrix. This polymer network structure not only improves the interfacial compatibility between polyurethane fibers and the NBR matrix but also reinforces the mechanical integrity and elasticity of the electrolyte while preserving effective ion transport pathways.

To visualize the mechanical enhancement of TVEE, we conducted in‐situ SEM testing to monitor its mechanical failure process under applied stress. As shown in Figure 1g, the TVEE underwent deformation when stress was applied, followed by crack initiation and eventual tearing as the stress increased. Remarkably, even when the film was torn, the polyurethane fibers remained firmly connected to the NBR matrix, indicating strong interphase adhesion and effective mechanical reinforcement. COMSOL simulations were also conducted to investigate stress distribution within the TVEE and NBR electrolyte membranes when subjected to stress. As shown in Figure 1h,i, compared to the stress concentration observed at the edge of the NBR electrolyte model, the TVEE membrane demonstrates significantly reinforced mechanical behavior. Intriguingly, the presence of the polyurethane fiber network enables efficient stress transmission, resulting in a more uniform stress distribution throughout the TVEE membrane.

**Figure 2:**
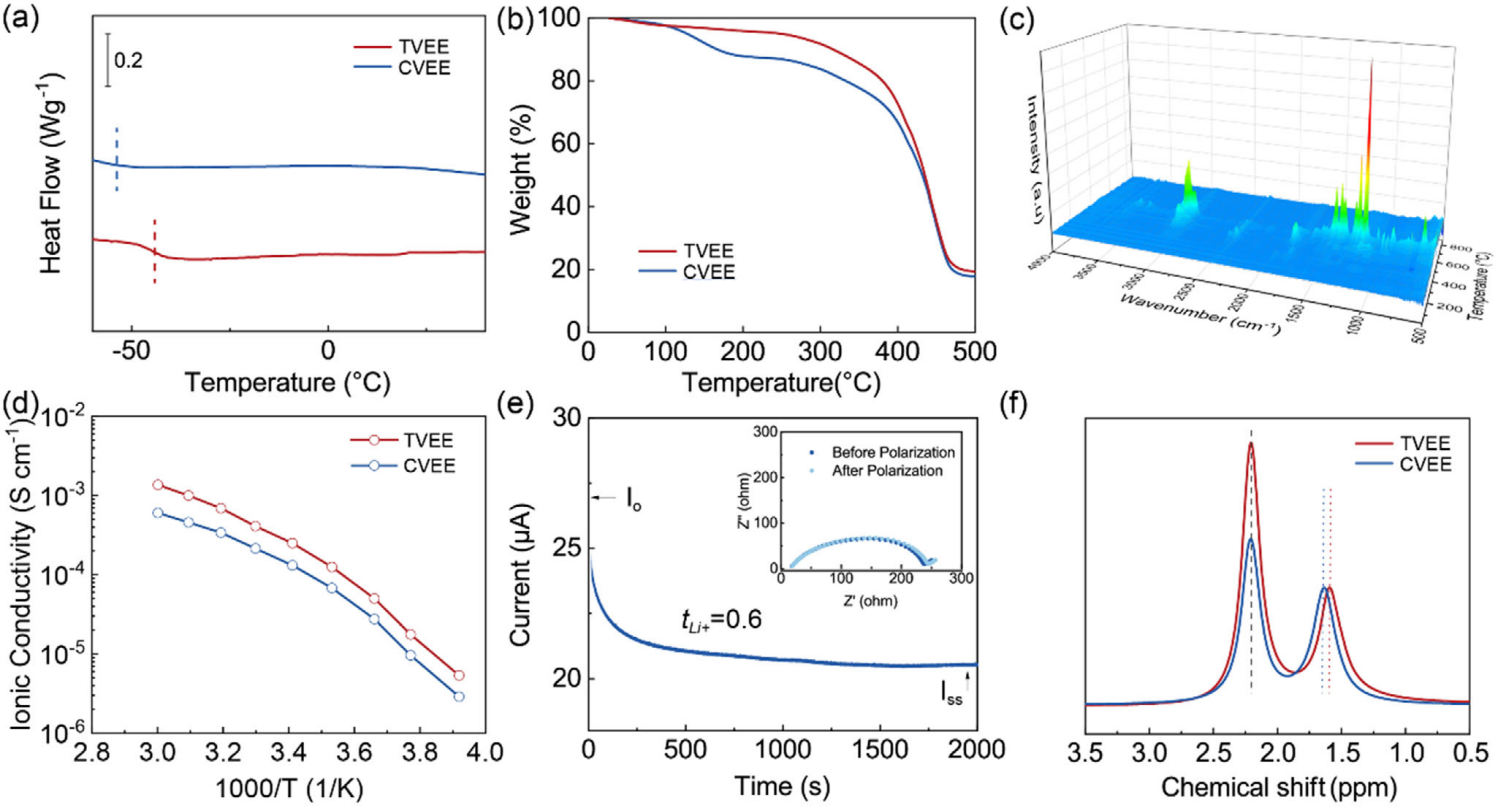
Lithium transport behaviors of elastomeric electrolytes. a) DSC curves and b) thermogravimetric curves for the TVEE and CVEE membranes. c) TG‐FTIR spectra of the TVEE membrane. d) Ionic conductivity‐temperature functions of the TVEE and CVEE membranes. e) The lithium‐ion transfer number test of the TVEE membrane. f) Solid‐state ^7^Li NMR spectra of the TVEE and CVEE membranes.

The thermodynamic properties of the elastomeric electrolytes were further investigated using differential scanning calorimetry (DSC) and thermogravimetric analysis (TGA). The DSC curves demonstrate that the TVEE membrane exhibits a low glass transition temperature of −48 °C (**Figure** **2**a), indicating a predominantly amorphous structure that facilitates lithium‐ion transport. The glass transition temperature (T_g_) of TVEE is slightly higher than that of the CVEE membrane (−52 °C). This can be attributed to the increased crosslink density resulting from the topological vulcanization process (Figure S13, Supporting Information). The absence of a separate polyurethane‐related T_g_ in the TVEE sample is likely due to the relatively low content of polyurethane and the interaction between polyurethane fibers and NBR, which may suppress or broaden the polyurethane T_g_ signal in the composite (Figure S14, Supporting Information). The TVEE membrane demonstrates superior thermal stability, exhibiting negligible weight loss before 260 °C (Figure 2b). A major decomposition peak appears at 380 °C, corresponding to the breakdown of the crosslinked NBR matrix, while a residual mass of 19.3% remains at 500 °C. This thermal stability significantly exceeds those of other elastomeric electrolytes reported in recent literature, which typically decompose below 300 °C.^[^ ^29^, ^30^ ^]^ Different from the TVEE membrane, the CVEE membrane shows an earlier onset of decomposition at 110 °C, attributed to the thermal decomposition of short‐chain sulfones and oligomers within the NBR matrix (Figures S15 and S16, Supporting Information). To further examine the thermal decomposition characteristics of the elastomeric electrolytes, thermogravimetric analysis coupled with Fourier‐transform infrared spectroscopy (TG‐FTIR) was performed (Figure 2c; Figure S17, Supporting Information). For the CVEE membrane, a minor S═O peak emerges at 130 °C, accompanied by a pronounced C═O signal at 1750 cm^−1^ during the initial stage of heat treatment. These signals are correlated with the decomposition of short‐chain sulfones and oligomers, indicating insufficient vulcanization of S─O bonds within the CVEE membrane. In contrast, the S═O signal was almost undetectable in the TVEE membrane, whereas the C═O and C─H signals emerged at 200 °C, which is indicative of the decomposition of long‐chain molecules. These observations are consistent with the XPS results, further confirming that most of the sulfur in the TVEE membrane serves as an efficient crosslinking agent by forming strong C─S bonds.

While the mechanical properties of elastomeric electrolytes are crucial, efficient lithium‐ion transport behaviors are equally essential for enhancing the overall electrochemical performance. To enhance lithium‐ion transport, 20 wt.% of LiTFSI was incorporated into the electrolytes and uniformly dissolved, as confirmed by the absence of the LiTFSI signal in the X‐ray Diffraction (XRD) patterns (Figure S18, Supporting Information). The ionic conductivities of both elastomeric electrolytes were evaluated at various temperatures to investigate their intrinsic lithium‐ion transport behavior. As depicted in Figure 2d, upon fitting the ionic conductivities to the Vogel‐Fulcher‐Tammann (VTF) equation, it is evident that both elastomeric electrolytes demonstrate characteristic solid‐state lithium‐ion transport behavior, indicating that ion mobility is thermally activated and structurally constrained by the matrix. The TVEE membrane demonstrates marginally higher ionic conductivity compared to the CVEE membrane. Moreover, the VTF activation energy for the TVEE membrane was calculated to be 0.031 eV, which is approximately one‐third of that for the CVEE membrane (0.097 eV) (Figure S19, Supporting Information), This improvement is primarily attributed to the topological vulcanization strategy, which induces spatially confined crosslinking within the NBR matrix. Such confined vulcanization ensures the formation of a robust elastomeric network while maintaining efficient lithium‐ion conduction through the NBR matrix within the topological network. This difference can be attributed to the negligible impact of topological vulcanization on the oxygen‐containing functional groups on the side chains of the matrix. Furthermore, the TVEE membrane exhibited a higher lithium‐ion transfer number of 0.6 compared to the CVEE membrane (Figure 2e; Figure S20, Supporting Information), which may be ascribed to the enhanced local segmental motions and continuous ion‐conducting pathway within the matrix.

^7^Li nuclear magnetic resonance (NMR) spectroscopy was utilized to further examine the local lithium‐ion environments within the elastomeric electrolytes. As depicted in Figure 2f, both the TVEE and CVEE membranes display two distinct peaks, each of which corresponds to different Li^+^ environments, namely, one associated with the skeleton and the other with the matrix. Specifically, the peak observed at 2.21 ppm is attributed to lithium ions that are strongly coordinated with molecular chains, thereby contributing negligibly to ion transport. In contrast, the left peak is attributed to the presence of highly mobile lithium ions, which arise from increased local disorder and weaker coordination with the molecular chains. Notably, the TVEE membrane demonstrated an upshifted signal at 1.59 ppm relative to 1.64 ppm in the CVEE membrane. This indicates a looser coordination environment, which consequently results in enhanced mobility of lithium ions.^[^ ^31^, ^32^ ^]^ These NMR results are consistent with the enhanced lithium‐ion transference number observed in the TVEE membranes, which can be ascribed to the efficient topological vulcanization within the NBR matrix.

**Figure 3:**
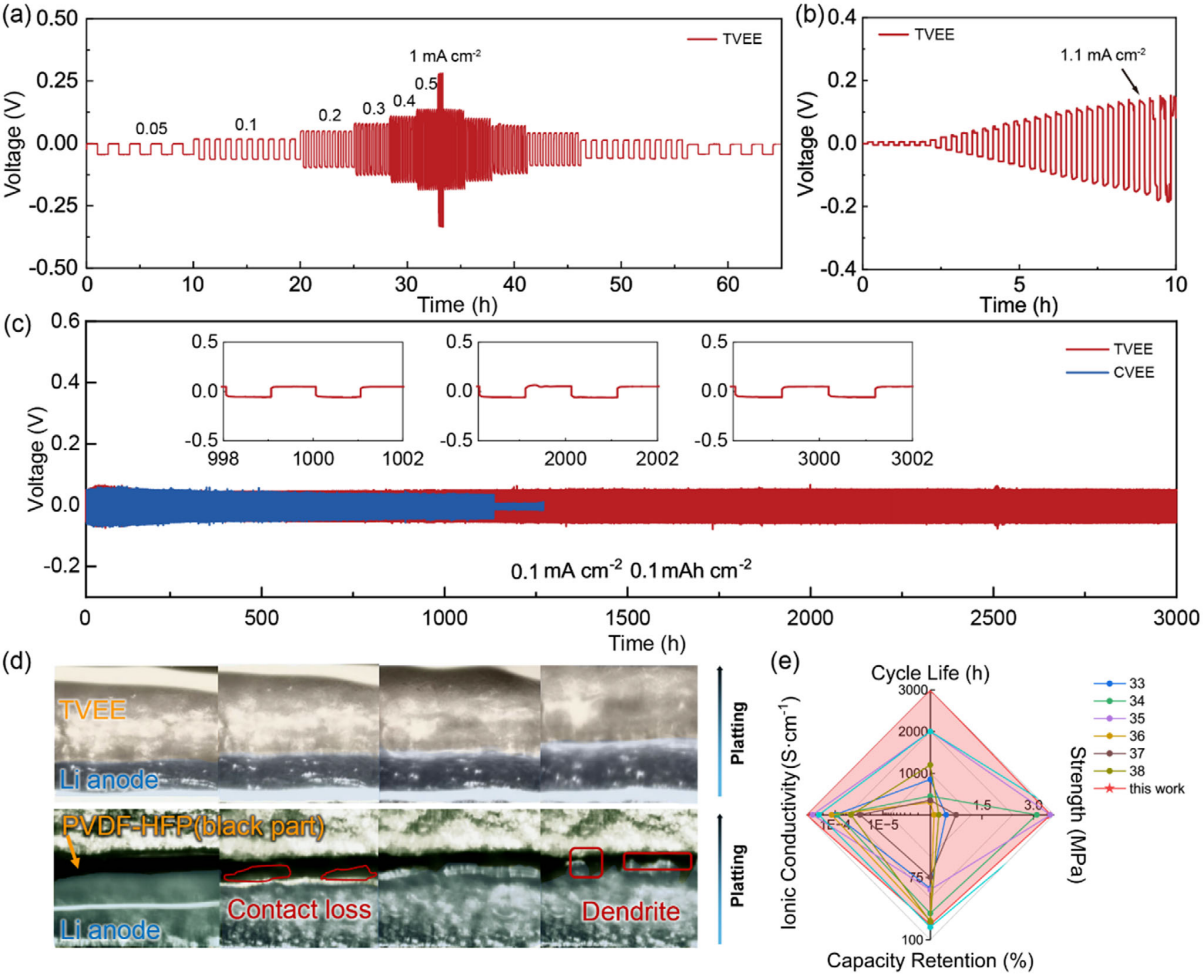
Electrochemical performance of the TVEE membrane. a) Rate performance and b) critical current density of the Li|TVEE|Li symmetric cells. c) Cycling performance of the Li|TVEE|Li and Li|CVEE|Li symmetric cells at 0.1 mA cm^−2^ for 0.1 mAh cm^−2^. d) In situ optical microscopy of lithium deposition and stripping behavior in the Li|TVEE|Li and Li|PVDF‐HFP|Li symmetric cells at 1 mA cm^−2^. e) Comparison of electrochemical stability and mechanical strength between TVEE and other solid polymer electrolytes.

Lithium symmetric cells were first assembled and subsequently tested to assess the electrochemical performance of the TVEE membrane. As depicted in **Figure** **3**a, the TVEE membrane exhibits superior rate performance across a wide range of current densities, varying from 0.05 to 1 mA cm^−2^. In contrast, the CVEEs exhibited significant polarization reactions and micro short circuits at high current densities. To further evaluate the safety and potential of TVEEs for high‐current‐density applications, a critical current density (CCD) test was performed. As depicted in Figure 3b, the TVEE membrane demonstrates a remarkable CCD of up to 1.2 mA cm^−2^, thereby underscoring its suitability for high‐power applications. Conversely, the CVEE membrane exhibits a relatively low CCD value of 0.8 mA cm^−2^ along with increased polarization under the same current density, which indicates its structural deficiencies and insufficient ionic transport pathways (Figure S21, Supporting Information). Furthermore, galvanostatic charge‐discharge tests were conducted to evaluate the long‐term cycling stability of the TVEEs. As depicted in Figure 3c and Figure S22 (Supporting Information), the TVEE membrane facilitates an extended cycle life with consistently low polarization over 3000 h at a current density of 0.1 mA cm^−2^ and over 600 h at a current density of 0.2 mA cm^−2^. In contrast, the CVEE exhibited unstable cycling performance, demonstrating a considerably shorter cycle life at 0.1 mA cm^−2^ and 0.1 mAh cm^−2^, and rapidly degrading under a higher current density of 0.2 mA cm^−2^ (Figure S23, Supporting Information). The inferior cycling performance of the CVEE membrane is likely attributed to the failure of interfacial contact between the CVEE membrane and the metallic lithium anode. This issue arises due to the insufficient mechanical strength of the CVEE membrane, which prevents it from accommodating long‐term and repeated volume changes, particularly under high current densities and capacities.

The interfacial impedance was initially characterized using electrochemical impedance spectroscopy (EIS) to assess the compatibility between the elastomeric electrolyte and the metallic lithium anode. The EIS plots of the lithium‐symmetric cells with TVEE indicate that their resistances exhibited only a slight increase during the initial cycles and maintained stable throughout the long‐term testing period. Moreover, the morphological characteristics of the lithium metal anodes after cycling were analyzed through SEM. As depicted in Figure S24 (Supporting Information), the metallic lithium anode cycled with the TVEE membrane demonstrates smooth and uniform surface morphology, indicative of stable and consistent contact during prolonged cycling. In contrast, the lithium anode cycled with the CVEE membrane exhibits a roughened surface, associated with non‐uniform lithium plating and the formation of “dead lithium”.

The XPS analysis of the metallic lithium anode cycled with the TVEE membrane further substantiates its exceptional interfacial compatibility. The analysis revealed that LiF is the predominant inorganic component within the solid electrolyte interphase layer, which contributes to enhanced stability and uniform lithium plating during cycling (Figure S25, Supporting Information). Additionally, the interfacial accommodation was further investigated via in situ optical microscopy. As shown in Figure 3d, when subjected to a high current density of 1 mA cm^−2^, the TVEE maintains intimate contact and elastic adaptation with the metallic lithium anode, ensuring uniform Li^+^ deposition without dendrite formation. In contrast, the non‐elastic polyvinylidene fluoride‐hexafluoropropylene (PVDF‐HFP) electrolyte cannot accommodate the volume changes during cycling, resulting in contact failure and eventually leading to the penetration of lithium dendrites. These observations highlight the ability of the TVEE membrane to accommodate volume changes and regulate interfacial ion distribution, even under high current densities. A comprehensive comparative analysis of the mechanical and electrochemical properties of state‐of‐the‐art organic solid electrolytes reported in the literature was conducted.^[^ ^33^, ^34^, ^35^, ^36^, ^37^, ^38^ ^]^ As illustrated in Figure 3e and Table S1 (Supporting Information), the TVEE electrolyte demonstrated exceptional overall performance, particularly in terms of symmetric battery cycle life, capacity retention in full cells, as well as mechanical strength and elastic properties.

**Figure 4:**
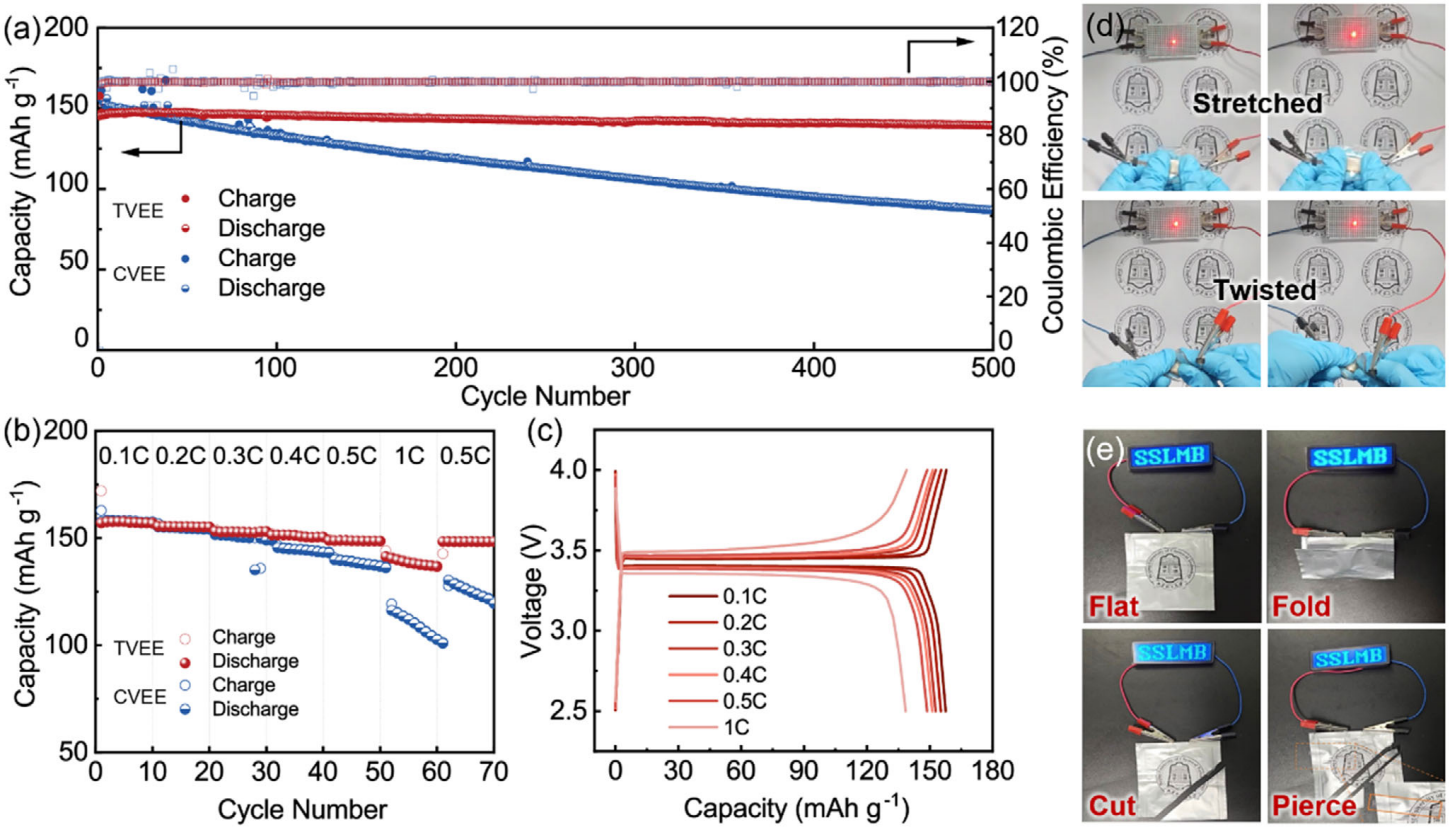
Full cell performance and pouch cell tests for the elastomeric electrolytes a) Cycling performance of the Li|TVEE|LFP and Li|CVEE|LFP cells at 0.5C. b) Rate capability and c) charge‐discharge profiles of the Li|TVEE|LFP and Li|CVEE|LFP cells at various rates. d) Optical images of a stretchable Li|TVEE|LFP battery powering an LED under 0% and 100% strain, as well as when twisted by 90° and 180°, respectively. e) Optical images of a Li|TVEE|LFP pouch cell lighting an LED panel undergoing folding, piercing and cutting.

To further verify the electrochemical performance of the TVEE membrane, full cells were assembled with metallic lithium anodes and LiFePO_4_ cathodes and subjected to galvanostatic charge‐discharge and rate capability tests. As shown in **Figure** **4**a, the full cell assembled with the TVEE membrane (Li|TVEE|LFP) exhibits outstanding cycle stability, achieving a long cycle life of 500 cycles with a high‐capacity retention of 95.7% at 0.5C. The excellent full‐cell cycle performance of the TVEE membrane can be attributed to the superior mechanical strength and durability of the TVEE membrane. In comparison, the full cell assembled with the CVEE membrane suffers rapid capacity decay, likely due to interfacial deterioration caused by contact failure between the electrolyte and electrodes. These findings are further supported by EIS data collected at various cycle numbers (Figure S26, Supporting Information). The EIS results indicate that the resistance of the full cell cycling with the TVEE membrane stabilized after a few initial cycles, further confirming the long‐term chemical and mechanical stability of the TVEE membrane during repeated lithium stripping and plating processes. The superiority of the TVEE membrane was also evident in rate performance tests (Figure 4b). In contrast to the significant capacity fade observed in the full cell with CVEE at high current densities, the full cell with TVEE delivers high capacities of 157, 155, 152, 148, and 138 mAh g^−1^ at current densities ranging from 0.1 to 1C, respectively (Figure 4c). The TVEE also exhibits superior compatibility with high‐voltage cathodes (Figure S27, Supporting Information). To further demonstrate the unique mechanical properties and superior electrochemical performance of the TVEE membrane, a stretchable battery was fabricated. The stretchable battery was fabricated by integrating a TVEE membrane, and stretchable electrodes, and encapsulating the assembly in polydimethylsiloxane. As depicted in Figure 4d, the stretchable battery reliably powered LED lights, even when subjected to a high strain of up to 200%. Furthermore, the stretchable battery demonstrated sustained functionality when being folded in half, twisted by 180°, or even rotated fully by 360°. This underscores TVEE membrane's exceptional performance, particularly in comparison to the majority of stretchable batteries that depend on flowable or liquid electrolytes.^[^ ^39^, ^40^ ^]^ Additionally, pouch cells were fabricated to investigate the practical applications and safety performance of the TVEE membrane in electronic devices. As illustrated in Figure 4e, the pouch cell effectively powered an LED panel under a variety of stress tests, including folding, cutting, and piercing. These findings demonstrate the reliability and significant potential of the TVEE membrane for integration into durable and safe electrical devices.

## 3 Conclusion

In summary, the elastomeric electrolyte has been successfully developed through the topological vulcanization strategy, achieving uniform crosslinking that results in an exceptional elongation at a break of 832%, robust mechanical strength of 3.51 MPa, and high ionic conductivity of 4 × 10^−4^ S cm^−1^ at room temperature. These properties surpass those of conventionally vulcanized counterparts, enabling stable cycling for up to 3000 h in lithium symmetric cells and demonstrating superior interfacial compatibility with metallic lithium anodes. The TVEE also displays remarkable durability and efficient ion transport, maintaining 95.7% capacity retention after 500 cycles at a current density of 0.5C in full cells. Furthermore, stretchable battery tests confirm its ability to withstand significant deformations without compromising electrochemical performance, highlighting its promising potential for applications in flexible and wearable electronics.

## Conflict of Interest

The authors declare no conflict of interest.

## Author Contributions

N.Y. and H.M. contributed equally to this work. N.Y. designed the experiments, analyzed the data, and wrote the original manuscript. H.M. conducted experiments and simulations with the help of N.Y., J.N., and Y.S. All the authors revised and edited the manuscript. F.W., J.N., and Y.S. conceived and supervised this project.

## Supporting information



Supporting Information

## Data Availability

The data that support the findings of this study are available in the supplementary material of this article.
