# Supplementary material for: Topological Vulcanization Strategy for Elastomeric Electrolytes with Enhanced Mechanical and Electrochemical Properties for Advanced Lithium Metal Batteries
Source: Adv Sci (Weinh). 2025 Jul 14;12(36):e06640. doi: 10.1002/advs.202506640 (PMC12463161; doi:10.1002/advs.202506640)
Supplement: Supplementary file 1 — Supporting Information [file ADVS-12-e06640-s001.docx]

**Supporting Information**

*Na Yang,^a,b^ Haotian Meng,^a,b^ DeCai Guo,^c^ Yongyi Song,^e^ Yongzheng Shi^d,*^ Jin Niu,^a,b,*^ Feng Wang^a,b,*^*

^a^State Key Laboratory of Chemical Resource Engineering, Beijing Key Laboratory of Electrochemical Process and Technology for Materials, Beijing University of Chemical Technology, Beijing 100029, P. R. China

^b^National Engineering Research Center for Fuel Cell and Hydrogen Source Technology, Beijing University of Chemical Technology, Beijing 100029, P. R. China

*^c^*Section 14, Dalian Research Institute of Petroleum and Petrochemicals, SINOPEC No. 96 Nankai Road, Tieshan Street, Lvshunkou District, Dalian, Liaoning Province, P. R. China

*^d^*College of Environmental Science and Engineering, North China Electric Power University, Beijing 102206, P. R. China

E-mail: shiyongzheng@ncepu.edu.cn; niujin@mail.buct.edu.cn; wangf@mail.buct.edu.cn

**Experimental section**

*Preparation of NBR-Based Electrolytes:* Firstly, 0.375 g of nitrile butadiene rubber (NBR, Baymod N XL 33.61, produced by LANXESS, containing 33 percent acrylonitrile) was dissolved in a mixed solution of 3 mL acetone and 6 mL dimethylbenzene to form a homogeneous precursor solution. Subsequently, a blend consisting of 5 wt.% 2,2’-azobis(2,4-dimethyl)valeronitrile (ABVN), 67 wt.% vinyl ethylene carbonate (VEC) monomer, and 67 wt.% 2,3,4,5,6-pentafluorostyrene was added, and the mixture was heated in an oil bath at 70 °C for 6 hours. Lithium bis(trifluromethanesulfonyl)imide (LiTFSI) was added after polymerization, followed by stirring for 2 hours at room temperature to dissociate the LiTFSI. The NBR-based electrolytes were thus obtained.

P*reparation of Sulfur-Loaded Polyurethane (PU) Membrane:* The polyurethane membrane was prepared using electrostatic spinning equipment (ET2535X, Ucalery). Firstly, a 25 wt.% sulfur-dissolved polyurethane solution was prepared by dissolving a specific amount of polyurethane in a tetrahydrofuran solution saturated with sulfur, and the mixture was stirred at room temperature for 12 hours. This solution was then loaded into a 10 mL disposable syringe equipped with a 21-gauge stainless steel needle. For the electrospinning process, the positive and negative voltages were set at +8 kV and -2 kV, respectively. Furthermore, the distance from the needle to the receiving roller covered with a conductive aluminum foil layer was maintained at 15 cm, the propulsion speed of the syringe was 0.1 mm/min, the ambient temperature was kept at 25°C, and the humidity at 20%. The sulfur-loaded polyurethane membrane obtained from electrospinning for 1 hour was then dried at room temperature for 6 hours.

*Fabrication of Topological Sulfide Electrolytes:* The topological sulfide electrolyte membrane was fabricated by separately casting the NBR-based electrolytes onto both sides of the sulfur-loaded polyurethane membrane. This was carried out on a polytetrafluoroethylene flat plate in an argon atmosphere. After drying at room temperature overnight in an argon atmosphere, the electrolyte was transferred to a vacuum at 60°C for 8 hours to completely remove the residual solvents. Lastly, the vacuum temperature was adjusted to 180°C, and the membrane was kept in the vacuum for 6 hours to enable gradient vulcanization of the NBR-based electrolyte at the interface with the sulfur-loaded polyurethane fiber. All corresponding matrices in this section were fabricated without adding additional LiTFSI

*Material characterization:* The morphology of electrolytes and anodes was characterized by SEM (JEOL, JSM-6701-F). Solid-state Proton nuclear magnetic resonance (^1^HNMR) and Lithium nuclear magnetic resonance (^7^Li NMR) spectroscopy were performed on JNM-ECZ 600R NMR spectrometers. X-ray diffraction (XRD) measurements were conducted using a Shimadzu XRD-7000 diffractometer with Cu Kα radiation. Differential scanning calorimetry (DSC) was performed using DSC-Q20 from TA Instruments at a heating rate of 10 °C/min in air. Thermogravimetric analysis (TGA) tests were carried out on a Netzsch X70 at a heating rate of 10 °C/min from 30 °C to 700 °C in N_2_ atmosphere. Attenuated total reflection Fourier transform infrared spectroscopy (ATR-FTIR) was performed on a Nicolet Nexus 670 spectrometer to characterize the specific chemical groups in the polymer electrolyte films. X-ray photoelectron spectroscopy (XPS) measurements were recorded on a Thermo Scientific ESCALAB 250. All the binding energies obtained in XPS spectra were calibrated using the C 1 s peak at 284.8 eV. DMA Q800 (TA Instruments) was used to test the tensile strength of the membranes at room temperature at a frequency of 1 Hz.

**Electrical measurements:** All the coin cells were assembled in a glove box filled with argon atmosphere (H_2_O < 0.01ppm, O_2_ < 0.01 ppm).

*Ionic conductivity (σ)*: The σ of the solid polymer electrolytes (SPEs) were characterized by d by electrochemical impedance spectroscopy (EIS) in the frequency range of 0.1 Hz to 10 MHz by a CHI 760E electrochemical workstation (Chenhua, Shanghai) at room temperature. The polymer electrolyte was sandwiched between two stainless steel (SS, 16 mm) blocking electrodes with an SS|SPE|SS configuration. The σ was calculated according to the following Eq. (S1):

$$\sigma=\frac{L}{R\cdot S}$$

(S1)

Where L (cm) IS the thickness of the polymer electrolyte, S (cm^2^) represents the area of the polymer electrolyte, and R (Ω) symbolizes the bulk ohmic resistance obtained by EIS.

*Ion transference number*: The lithium-ion transference number (*t*_Li+_) of the SPE was obtained by combining AC impedance and DC polarization techniques using a Li symmetry cell at room temperature. Before measurement, the coin cell was first cycled at 0.1mA cm^-2^ and 0.1mAh cm^-2^ for 20 h to form a stable interface between the polymer electrolyte and electrode. EIS measurement was conducted before and after polarization. *t*_Li+_ was calculated according to the following Eq. (S2)

$$t_{Li+}=\frac{I_{s}Rb_{s}(\Delta V-I_{o}R_{o})}{I_{o}Rb_{o}(\Delta V-I_{s}R_{s})}$$

(S2)

Where Io and Iss are the initialed steady-state current values, respectively. ΔV represents the polarization potential which is 10 mV, Rb_o_ and Rb_ss_ represent the initial and steady-state values of the bulk resistances, respectively.

*Electrochemical stability window*: The electrochemical stability of the SPEs was investigated by linear sweep voltammetry (LSV) on a CHI 760E electrochemical workstation (Chenhua, Shanghai). The Li|SPE|SS coin cell was measured over a positive potential range from open circuit potential to 6.0 V at a scan rate of 0.1 mV s^−1^ at room temperature.

*Critical current density:* Critical current density was conducted with symmetrical Li|SPE|Li cells. Cells were cycled at low current density for the first few cycles for activation and then cycled at a varied current density from 0.05 to 1 mA cm^-2^ for 30 min per step at 60 °C.

*COMSOL simulation:* Solid mechanics simulations were conducted using COMSOL Multiphysics 6.3, based on the principles of solid mechanics. To simplify the force model, a cuboid was set with two layers of fibers which intersect perpendicular to each other as surfaces. A load of 5 mN was applied along the normal direction of the Y-axis on the outer side of the assembly. After establishing the mesh, a steady-state study was conducted in the mechanical field. Based on the principles of solid mechanics, the stress distribution characteristics of the membrane under tensile conditions were simulated.


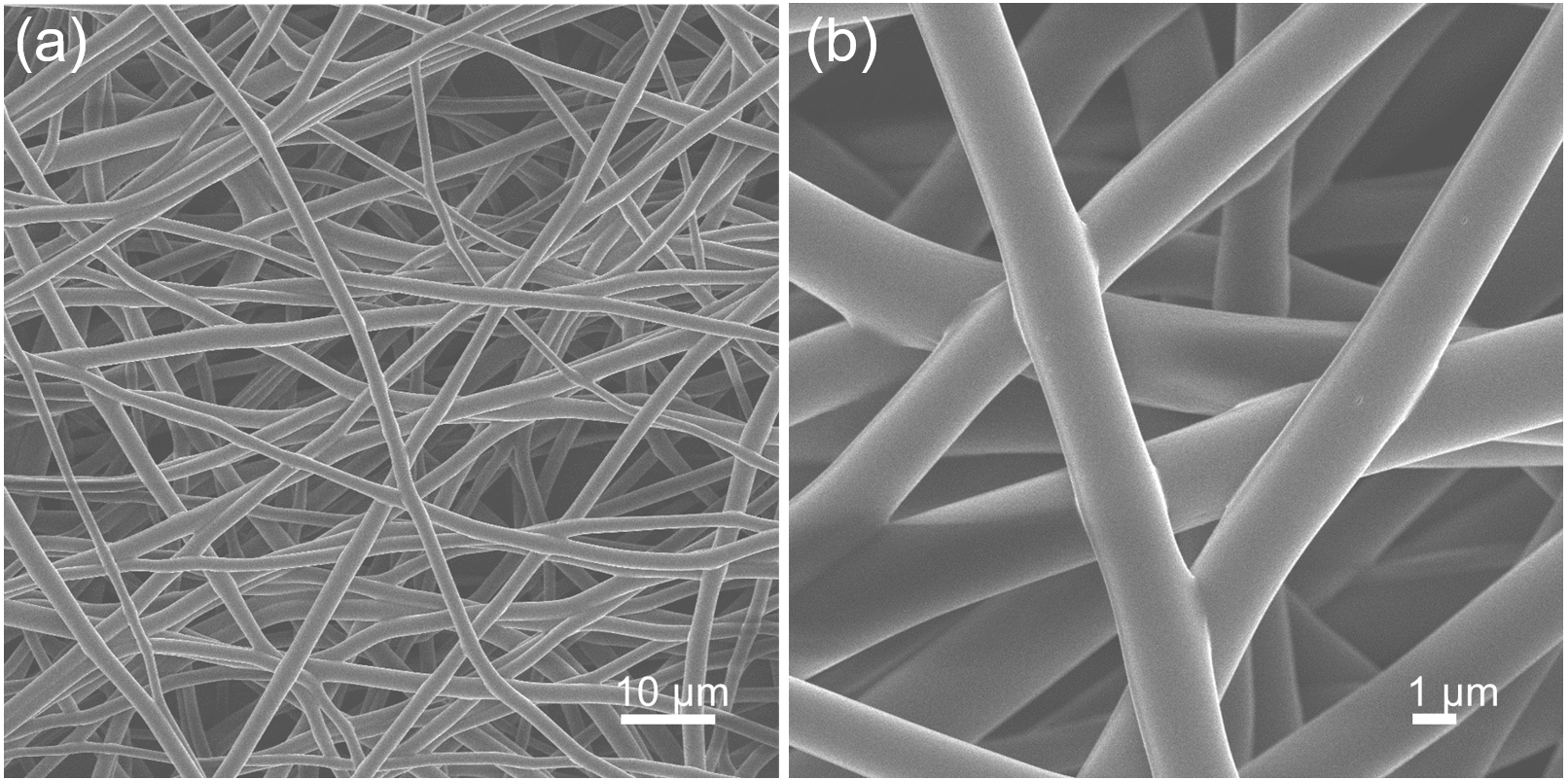


1. SEM images of the S-polyurethane fiber film.


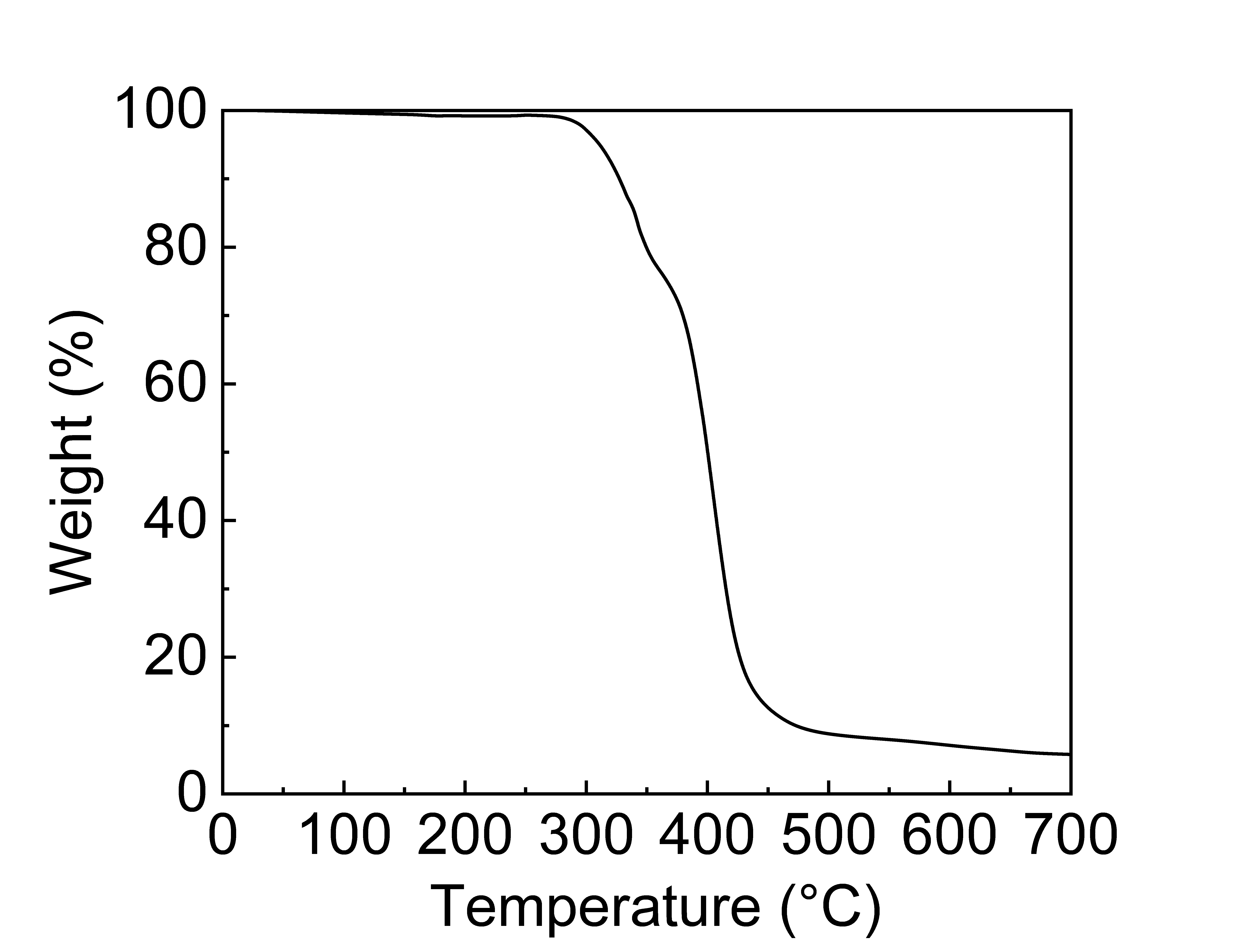


1. TGA curve of Polyurethane fiber membrane.


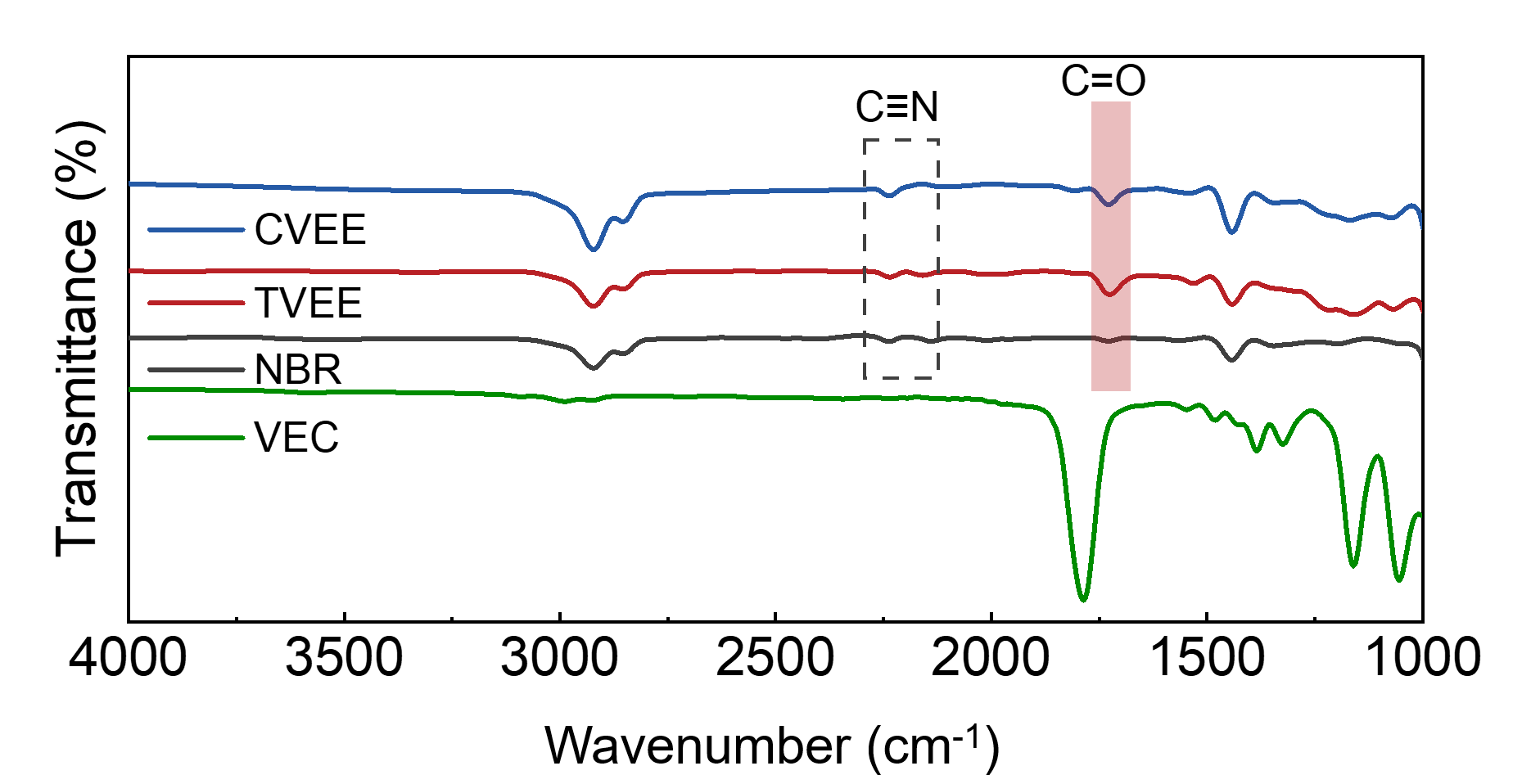


1. Amplified FTIR spectra of the CVEE, TVEE, NBR electrolyte and VEC.


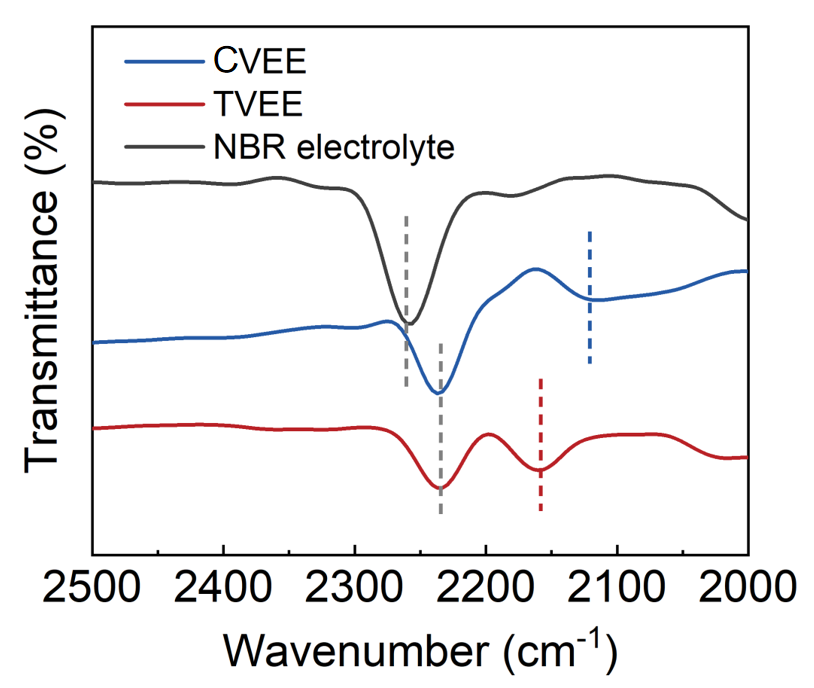


1. Amplified FTIR spectra of the CVEE, TVEE and NBR electrolyte.


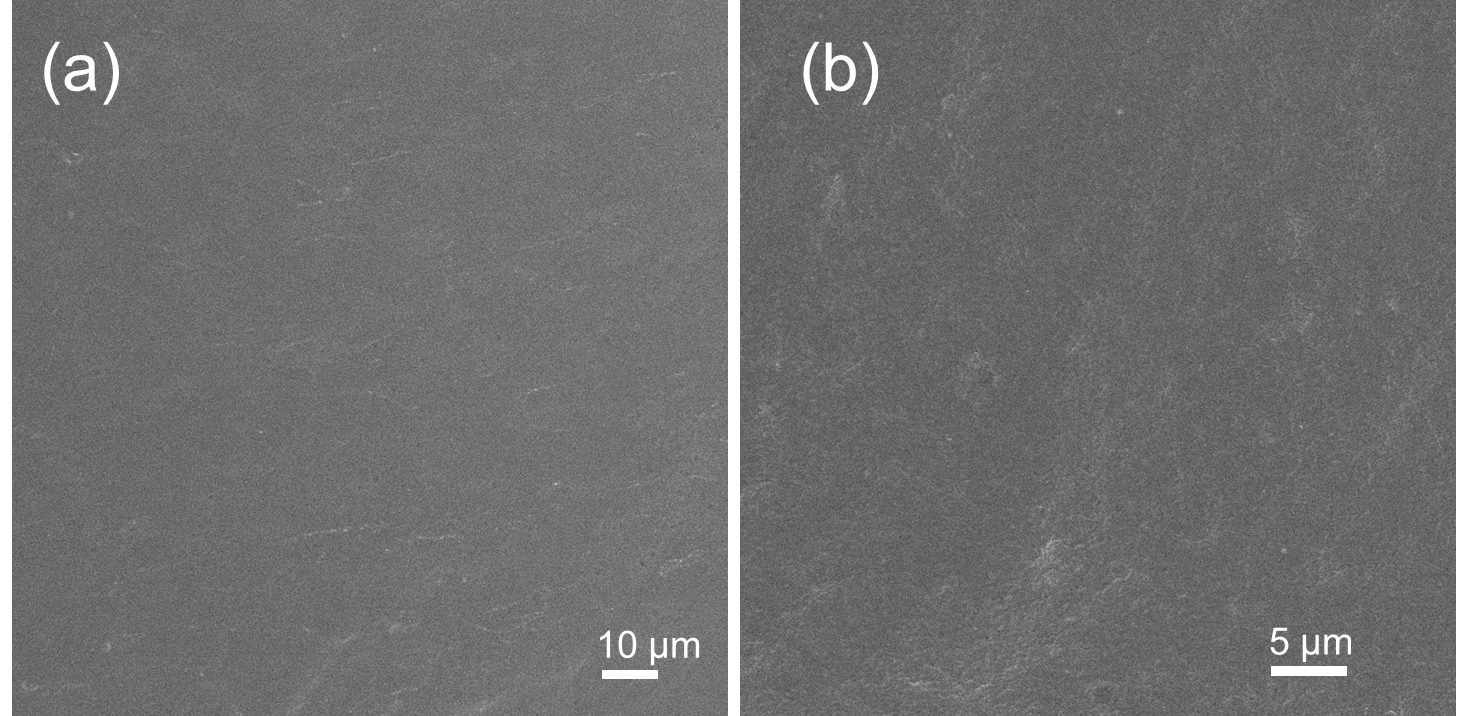


1. Top-view SEM images of the TVEE.


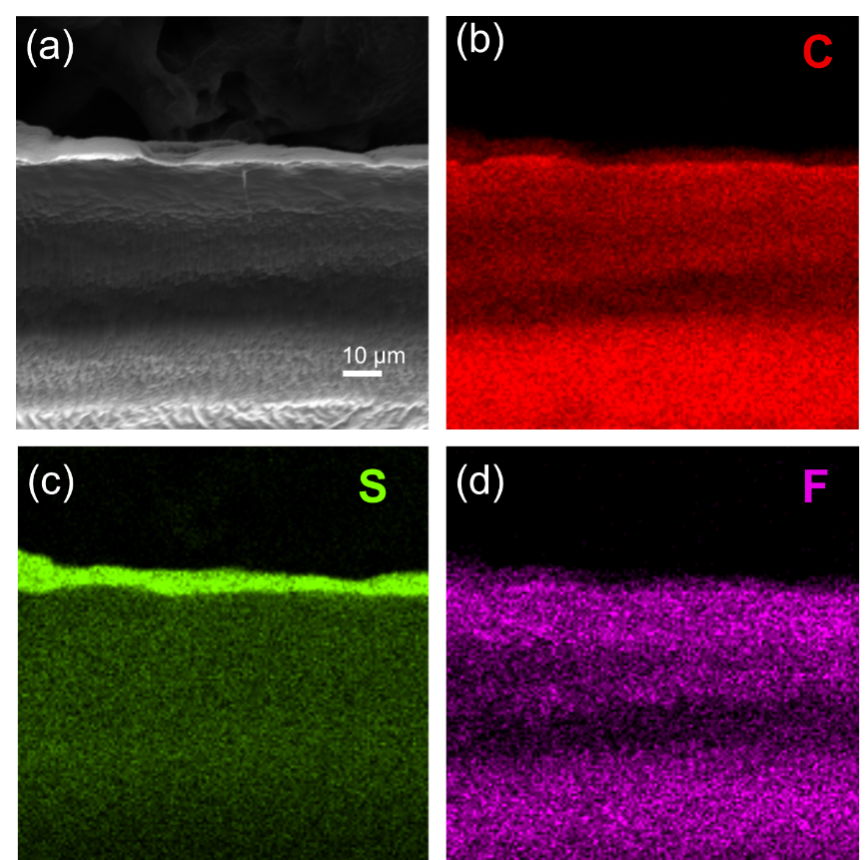


1. (a) The cross-section SEM image of the TVEE matrix and (b-d) corresponding elemental mapping images of C, S, and F.


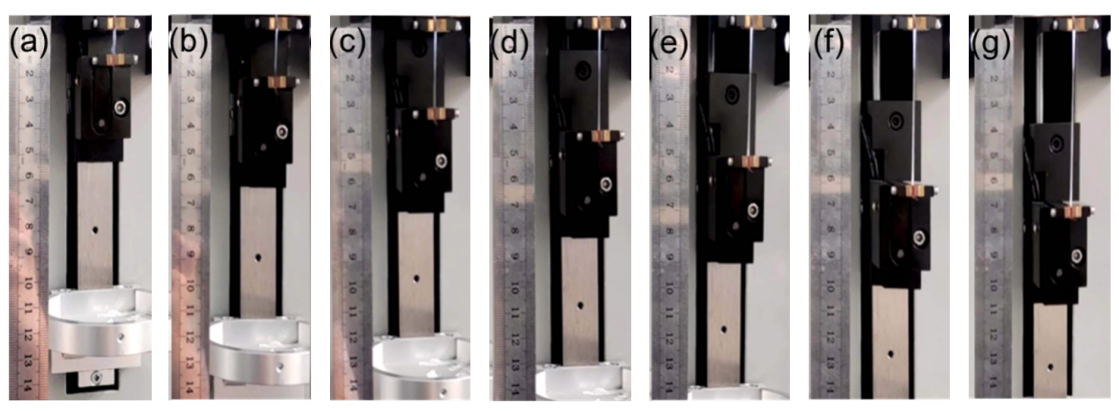


1. (a-g) The optical images of TVEE electrolyte under stress.


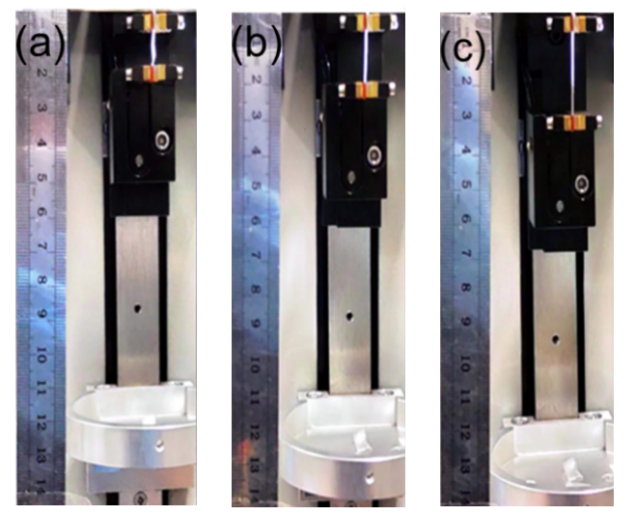


1. (a-c) The optical images of CVEE electrolyte under stress.


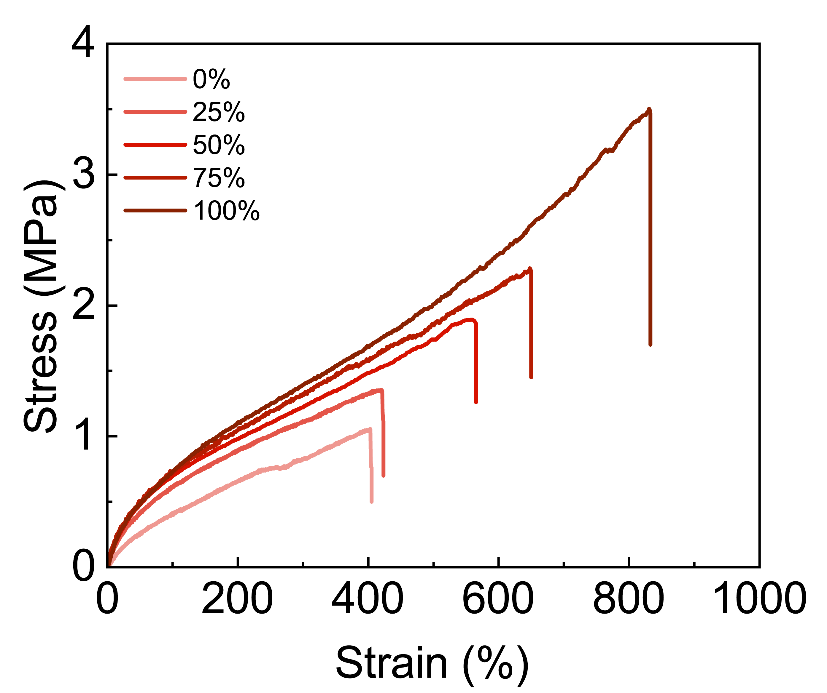


1. Stress-strain curves of TVEE with different degree of topological vulcanization.


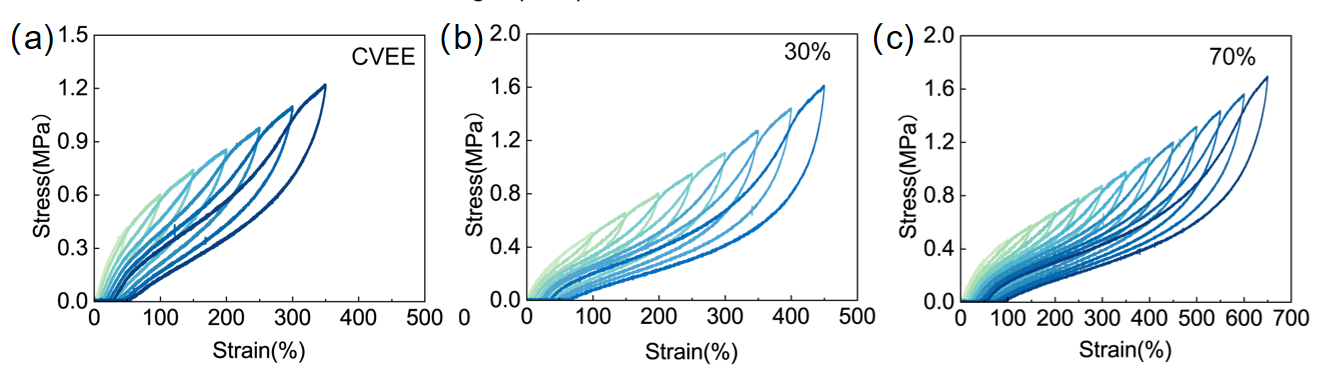


1. Stress-strain cycling curves of the (a) CVEE, (b) 30% vulcanized and (c) 70% vulcanized TVEE electrolyte membranes.


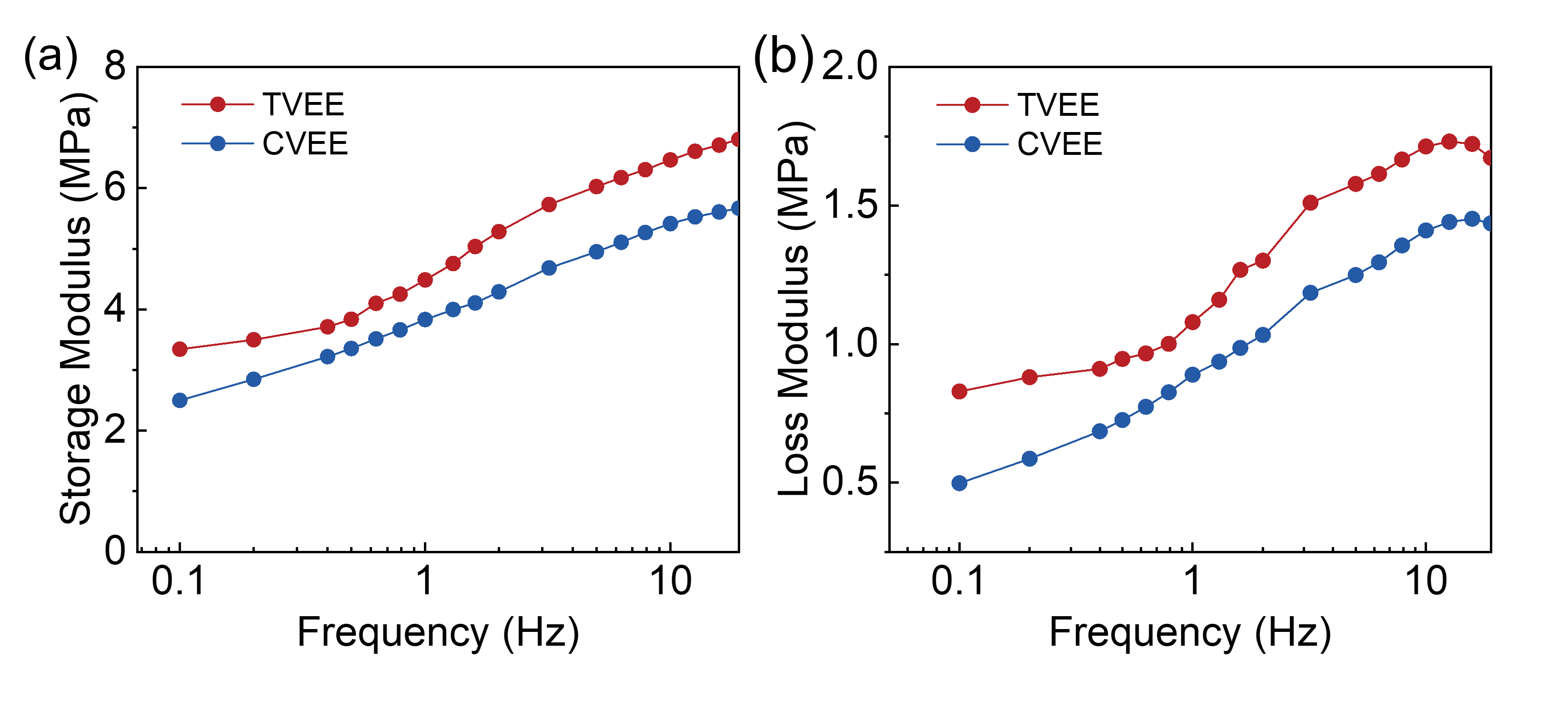


1. Frequency sweep curves of TVEE and CVEE (a) storage modulus and (b) loss modulus.


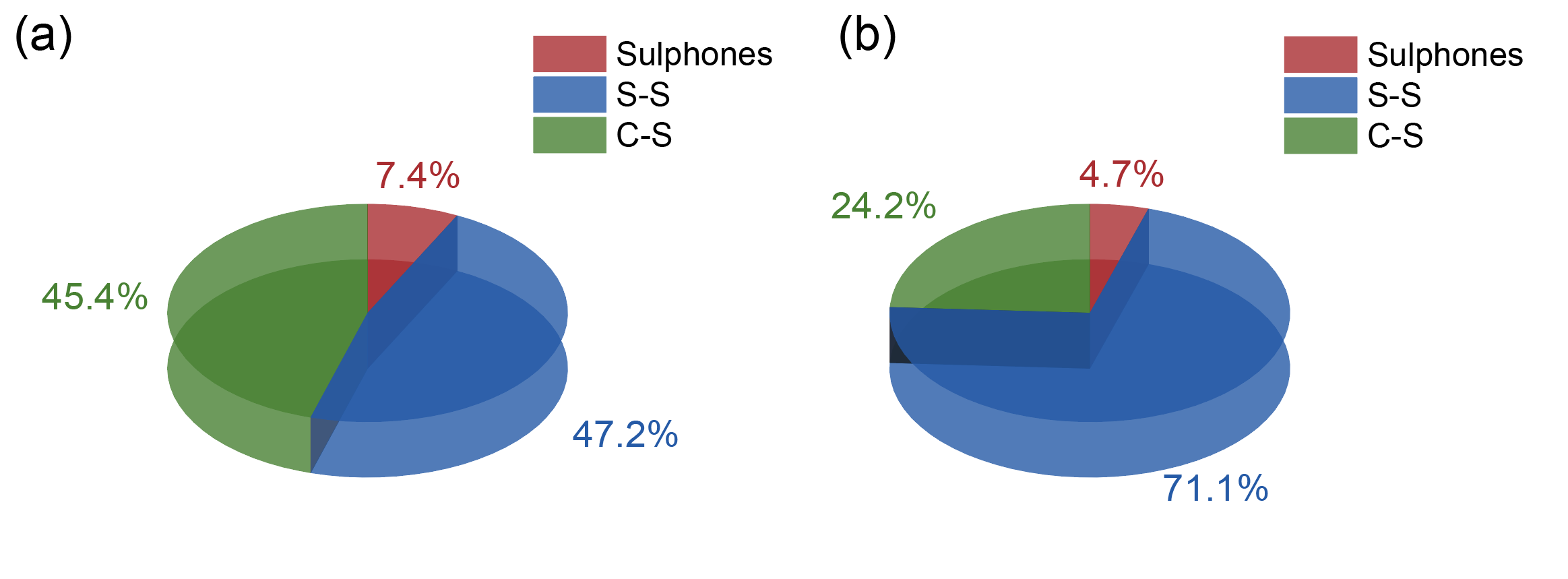


1. XPS S 2p peak area analysis for (a) TVEE and (b) CVEE.


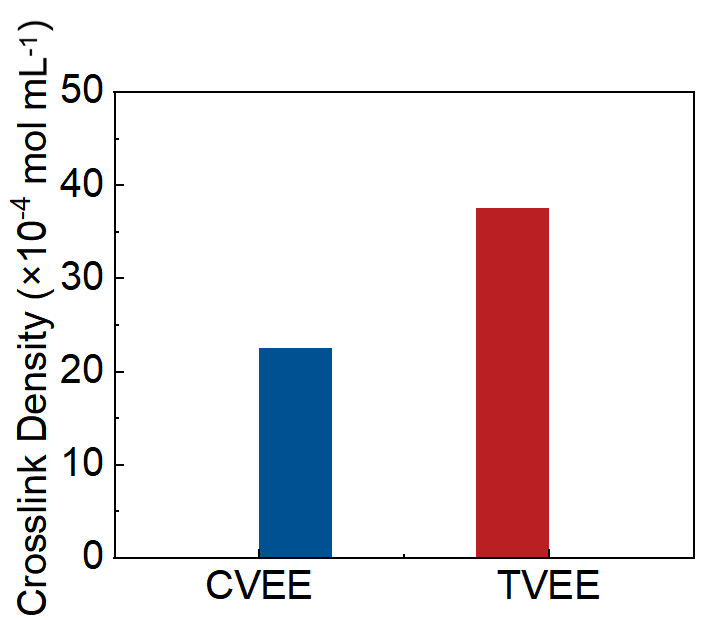


1. Crosslink density of CVEE and TVEE membranes.


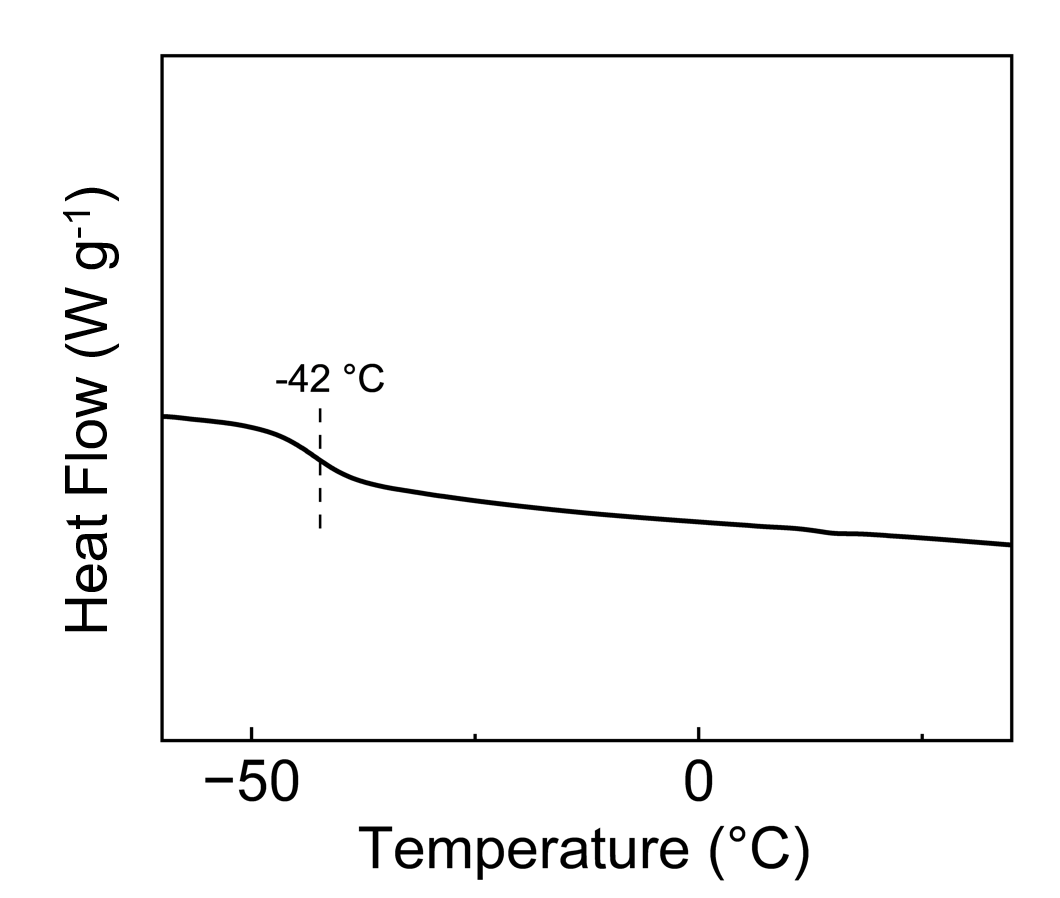


1. DSC curve for the polyurethane fiber membrane.


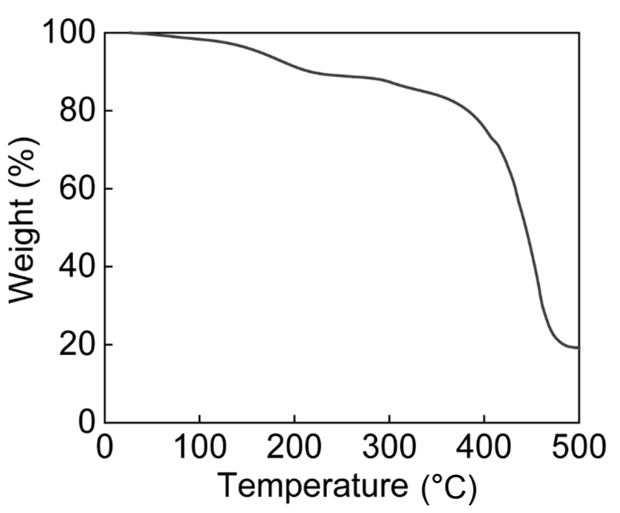


1. TGA curves of NBR electrolyte.

1. Thermogravimetric analysis and corresponding derivative of thermogravimetric curves for the TVEE and CVEE membranes.


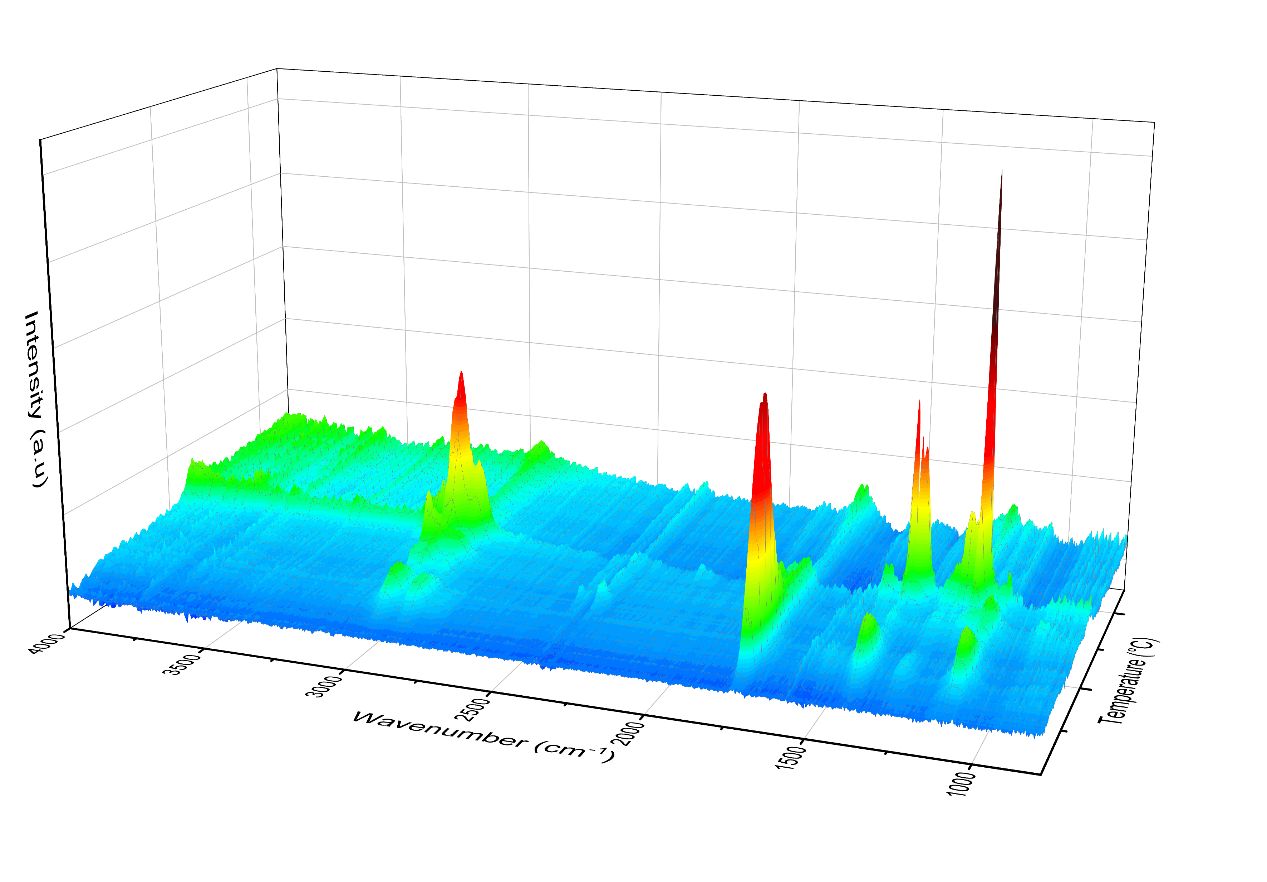


1. TG-FTIR patterns of CVEE heated in an Ar atmosphere at a heating rate of 10 °C min^-1^.

1. XRD patterns of different types of solid elastic electrolytes and LiTFSI.


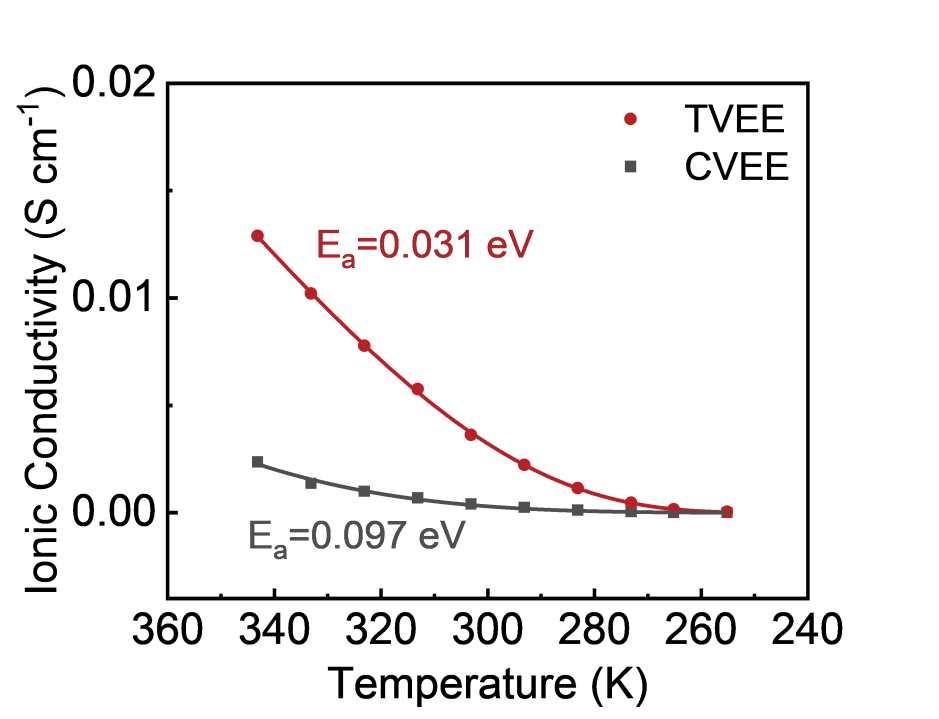


1. VTF fitting and calculated activation energy of the TVEE and CVEE membranes.

1. The *t*_Li_^+^ test of the CVEE.


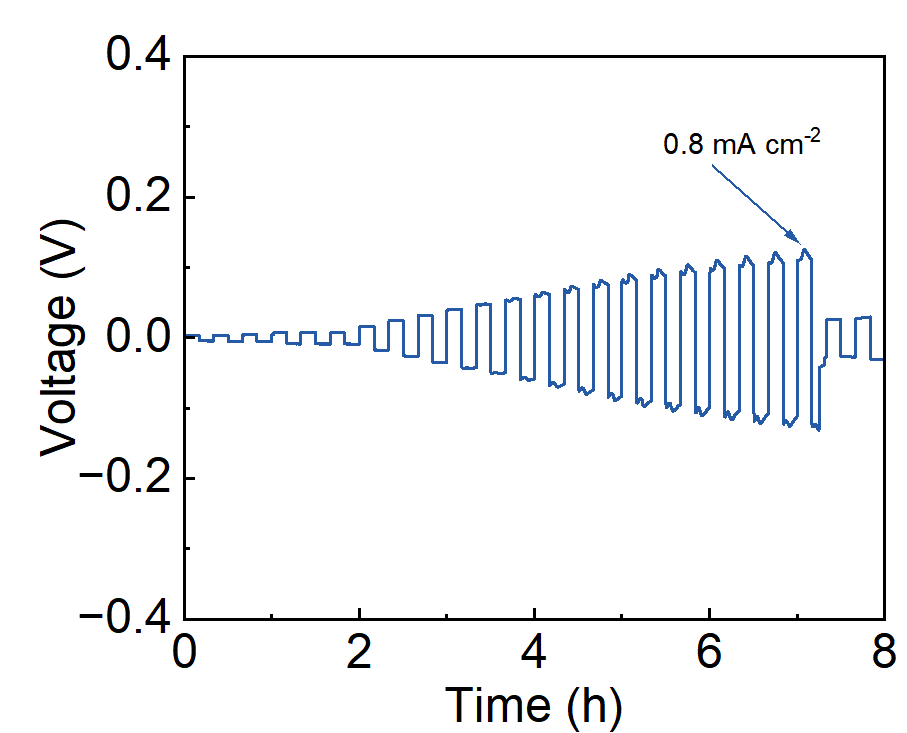


1. Critical current density test of the Li|CVEE|Li symmetric cell.


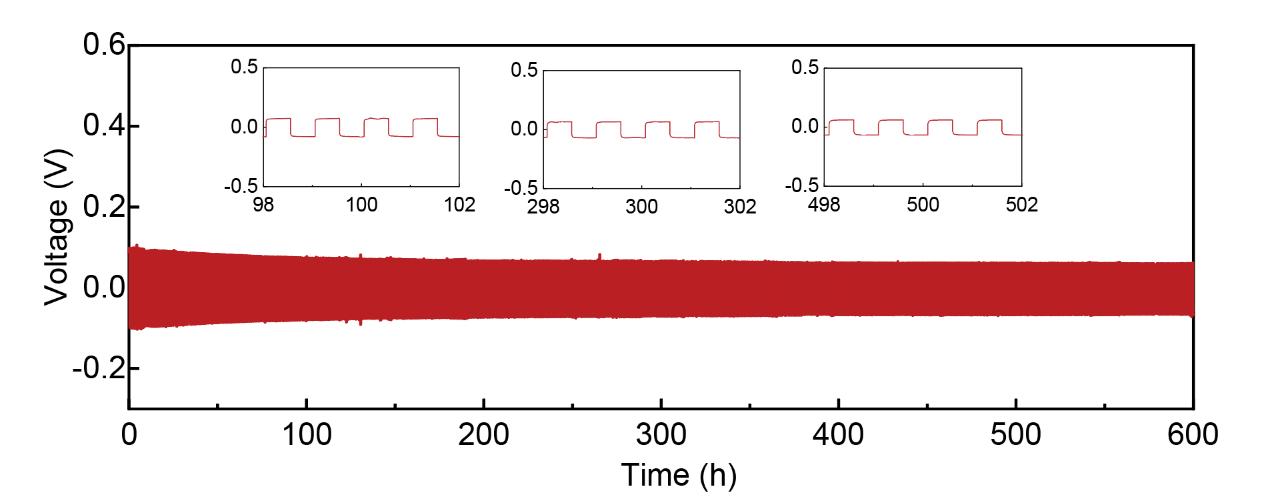


1. Cycling performance for Li symmetric cell with the TVEE at current densities of 0.2 mA cm^-2^and a capacity of 0.1 mAh cm^-2^.


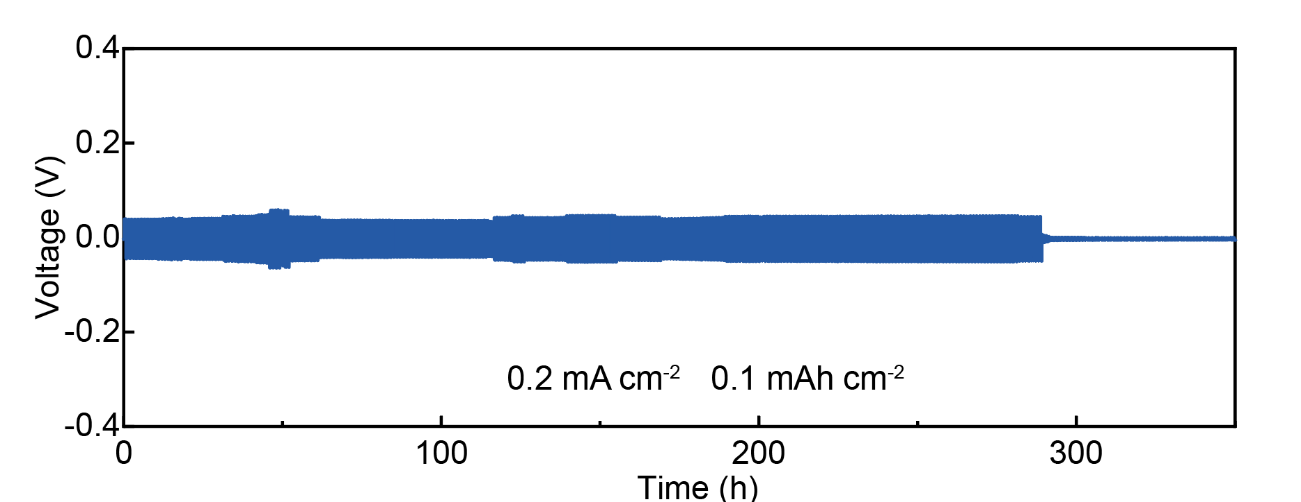


1. Cycling performance for Li symmetric cell with the CVEE at current densities of 0.2 mA cm^-2^and a capacity of 0.1 mAh cm^-2^.


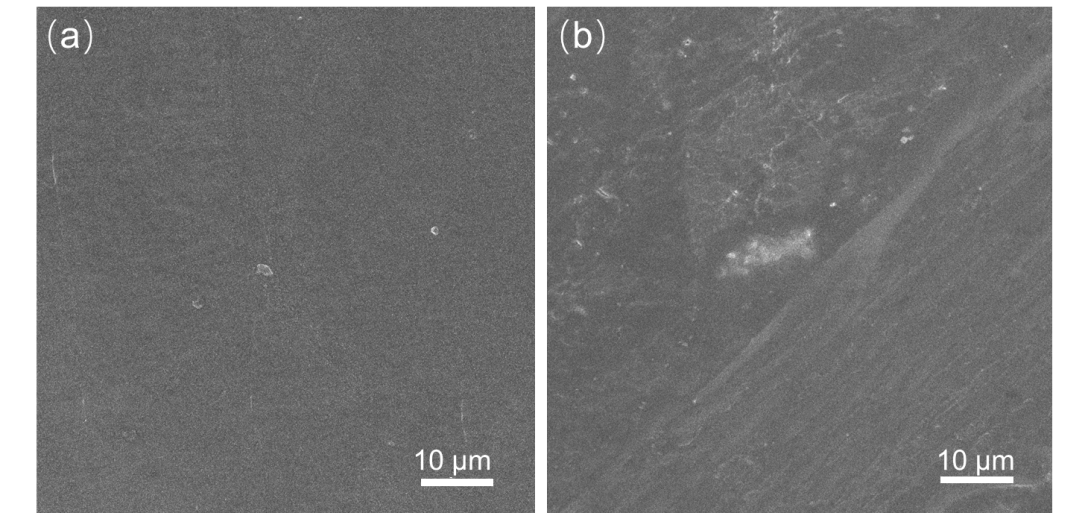


1. SEM images of the cycled lithium anodes with the (a) TVEE and (b) CVEE.


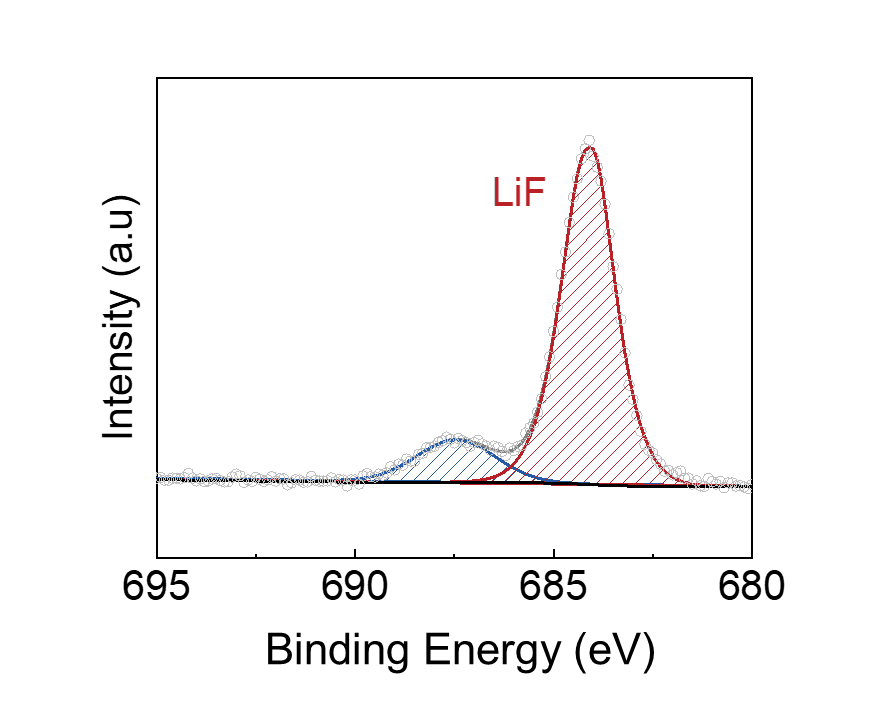


1. XPS spectra of F 1s for the cycled lithium anode with the TVEE by Ar^+^ sputtering for 200s.

1. The resistance of the full cell cycled with the TVEE membrane stabilized after a few initial cycles.


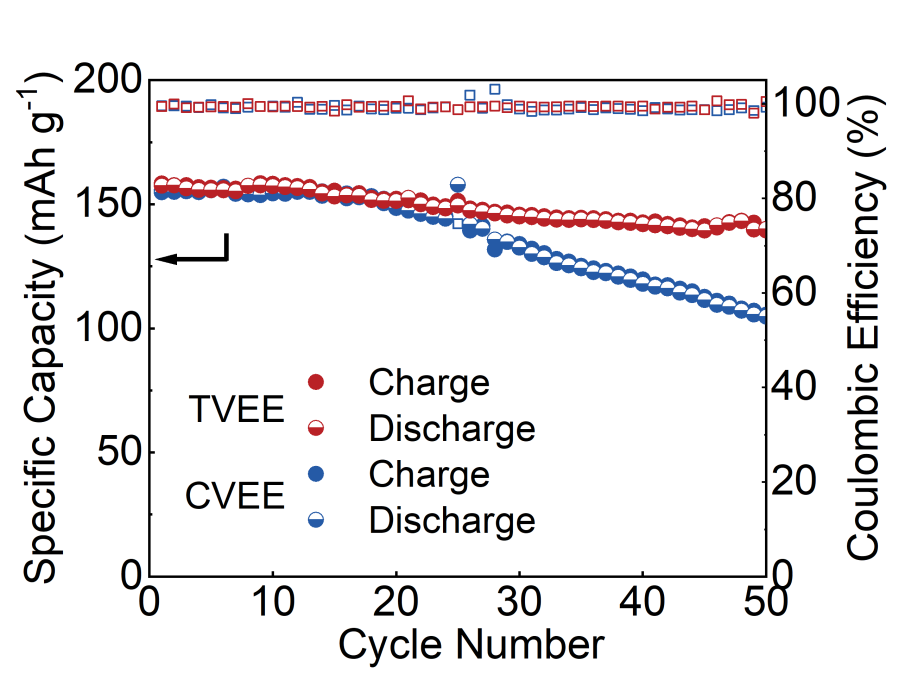


1. Full cell cycling performance of the Li|TVEE|NCM811 and Li|CVEE|NCM811 cells at 0.5C.

**Table S1.** Comparison of the ionic conductivity, lithium-ion transference number, strain, strength and cycle life of the TVEE electrolyte with previous work.

| **Electrolyte** | **Ionic conductivity (S cm^-1^)** | **Li^+^ transference number** | **Strain (%)** | **Strength (MPa)** | **Cycle life (h)** |
| --- | --- | --- | --- | --- | --- |
|  |  |  |  |  |  |
|  |  |  |  |  |  |
| TVEE | 4×10^-4^ | 0.6 | 832 | 3.51 | 3000 |
| CVEE | 1.5×10^-4^ | 0.438 | 474 | 1.8 | 1100 |
| PEA-IL^[30]^ | 1×10^-4^ | - | 5200 | 0.05 | 800 |
| PEO/PI^[31]^ | 6×10^-5^ | 0.265 | 40 | 3.1 | 450 |
| Poly(DOL-TTE)  -LP^[32]^ | 3×10^-4^ | 0.35 | 110 | 3.3 | 2000 |
| PEL-0.1^[33]^ | 1.19×10^-4^ | 0.36 | 1000 | 0.11 | 300 |
| HPAE^[34]^ | 3×10^-5^ | 0.67 | 40 | 0.75 | 350 |
